# Hedgehog Promotes Neovascularization in Pancreatic Cancers by Regulating Ang-1 and IGF-1 Expression in Bone-Marrow Derived Pro-Angiogenic Cells

**DOI:** 10.1371/journal.pone.0008824

**Published:** 2010-01-21

**Authors:** Kazumasa Nakamura, Junpei Sasajima, Yusuke Mizukami, Yoshiaki Sugiyama, Madoka Yamazaki, Rie Fujii, Toru Kawamoto, Kazuya Koizumi, Kazuya Sato, Mikihiro Fujiya, Katsunori Sasaki, Satoshi Tanno, Toshikatsu Okumura, Norihiko Shimizu, Jun-ichi Kawabe, Hidenori Karasaki, Toru Kono, Masaaki Ii, Nabeel Bardeesy, Daniel C. Chung, Yutaka Kohgo

**Affiliations:** 1 Division of Gastroenterology and Hematology/Oncology, Department of Medicine, Asahikawa Medical College, Asahikawa, Japan; 2 Department of General Medicine, Asahikawa Medical College, Asahikawa, Japan; 3 Department of Animal Facility, Asahikawa Medical College, Asahikawa, Japan; 4 Department of Cardiovascular Regeneration and Innovation, Asahikawa Medical College, Asahikawa, Japan; 5 Division of Gastroenterological and General Surgery, Department of Surgery, Asahikawa Medical College, Asahikawa, Japan; 6 Group of Vascular Regeneration Research, Institute of Biomedical Research and Innovation, Kobe, Japan; 7 Massachusetts General Hospital Cancer Center and Harvard Medical School, Boston, Massachusetts, United States of America; 8 Gastrointestinal Unit, Massachusetts General Hospital and Harvard Medical School, Boston, Massachusetts, United States of America; Ohio State University, United States of America

## Abstract

**Background:**

The hedgehog (Hh) pathway has been implicated in the pathogenesis of cancer including pancreatic ductal adenocarcinoma (PDAC). Recent studies have suggested that the oncogenic function of Hh in PDAC involves signaling in the stromal cells rather than cell autonomous effects on the tumor cells. However, the origin and nature of the stromal cell type(s) that are responsive to Hh signaling remained unknown. Since Hh signaling plays a crucial role during embryonic and postnatal vasculogenesis, we speculated that Hh ligand may act on tumor vasculature specifically focusing on bone marrow (BM)-derived cells.

**Methodology/Principal Findings:**

Cyclopamine was utilized to inhibit the Hh pathway in human PDAC cell lines and their xenografts. BM transplants, co-culture systems of tumor cells and BM-derived pro-angiogenic cells (BMPCs) were employed to assess the role of tumor-derived Hh in regulating the BM compartment and the contribution of BM-derived cells to angiogenesis in PDAC. Cyclopamine administration attenuated Hh signaling in the stroma rather than in the cancer cells as reflected by decreased expression of full length Gli2 protein and Gli1 mRNA specifically in the compartment. Cyclopamine inhibited the growth of PDAC xenografts in association with regression of the tumor vasculature and reduced homing of BM-derived cells to the tumor. Host-derived Ang-1 and IGF-1 mRNA levels were downregulated by cyclopamine in the tumor xenografts. *In vitro* co-culture and matrigel plug assays demonstrated that PDAC cell-derived Shh induced Ang-1 and IGF-1 production in BMPCs, resulting in their enhanced migration and capillary morphogenesis activity.

**Conclusions/Significance:**

We identified the BMPCs as alternative stromal targets of Hh-ligand in PDAC suggesting that the tumor vasculature is an attractive therapeutic target of Hh blockade. Our data is consistent with the emerging concept that BM-derived cells make important contributions to epithelial tumorigenesis.

## Introduction 

Pancreatic ductal adenocarcinoma (PDAC) is the fourth leading cause of cancer-related death in the United States and fifth in Japan, and its overall 5-year survival rate is only 5.0–9.7% [1], [2]. Gemcitabine-based chemotherapy for locally advanced or metastatic PDAC has only modest activity with a small survival benefit [3]. The identification of new molecular targets for PDAC to overcome the dismal prognosis is therefore necessary. Thus far, a range of targeted therapies against EGFR [4], Ras/MEK [5] and VEGF [6] have failed to improve survival significantly in clinical trials.

Aberrant activation of the hedgehog (Hh) pathway has more recently been recognized as one of the mediators of PDAC development and is therefore considered to be a promising target for therapy [7], [8]. Hh is a morphogen required for proper pattern formation during embryogenesis. Elevated expression of Hh-related proteins, sonic hedgehog (Shh) or indian hedgehog (Ihh), impairs pancreatic morphogenesis with increases in mesenchyme and decreases in the epithelial compartment [9], indicating that tight regulation of the overall level of Hh activity is crucial for normal pancreatic development. Since Shh is misexpressed not only in PDAC but also in the precursor lesions pancreatic intraepithelial neoplasm (PanIN) [8] and intraductal papillary mucinous neoplasia [10], dysregulation of this pathway may play a role during early stages of tumorigenesis. A number of Hh-target genes, such as Bmp [11], FOXM1 [12], and PMP22 [13], are overexpressed in PDAC, and there is cross-talk between oncogenic pathways including MAPK/ERK, PI3K/Akt [14], Wnt [15], TGFβ/BMP [16] and Hh signaling. Among the molecules that participate in the Hh signaling, Smoothened (Smo) has been recognized as a key mediator of the signaling; it is one of the receptor components for Hh ligand and converts Gli2 into a transcriptional activator [17]. Thus, Smo is a particularly promising target for anti-cancer therapy [18]. Cyclopamine, a natural steroid alkaloid, has strong inhibitory effects against Smo [18] and treatment with cyclopamine in the experimental setting inhibits growth of many cancers [19], [20], [21]. Cyclopamine derivatives with various structural motifs have been generated and show promise for possible clinical use [22].

Specifically, several studies have demonstrated significant growth inhibition of PDAC *in vivo* by Hh pathway blockade [7], [23]. The tumor growth inhibitory mechanism has been thought to be primarily mediated through blocking an autocrine loop that might be required for proliferation [7], survival [8] and motility [21] of cancer cells. However, Smo deletion in the pancreatic epithelium does not impair tumorigenesis in a mouse model of PDAC [24], and Hh ligands do not induce transcriptional reporters of the pathway in PDAC lines. Moreover, transgenic expression of an activated Smo allele in the pancreatic epithelium fails to induce Smo pathway target genes or to induce neoplastic change [25]. Consequently, it has been speculated that Hh signaling might play a paracrine role within the tumor microenvironment, in epithelial cancers that lack mutations in Hh signaling components. Indeed, studies performed by us and others have defined crucial roles of cancer cell derived Hh on cell types within tumor stromal compartments [26], [27], [28]. Despite these findings, the precise mechanisms by which some tumor cells are insensitive to Hh inhibition and the stromal cell type(s) that are responsive to Hh signaling remained unknown. We therefore undertook studies to elucidate these mechanisms.

## Methods

### Cell Culture, Protein Analysis, and RNA Analysis

Human PDAC cell lines were obtained from ATCC, Health Science Research Resources Bank (Osaka, Japan) and Cell Resource Center for Biochemical Research (Sendai, Japan). BMMNC isolation, proliferation assays, co-culture, migration assays, immunoblotting, and qPCR are described in Methods S1. Primer sequences are summarized in **Table S1**.

### Animals, Bone Marrow Transplantation (BMT) and Immunohistochemistry

Therapeutic studies with cyclopamine were performed with nude mice xenografts as described [7]. BMT procedures are provided in Methods S1. Protocols for animal experiments were approved by the Asahikawa Medical Collage Institutional Animal Care and Use Committee.

Tissues were processed as reported previously [26]. Antibodies and conditions for immunohistochemistry are presented in Methods S1.

### Cellular, Molecular, and Statistical Analyses

Detailed descriptions of procedures are provided in Methods S1.

## Results

### *In Vivo* Effects of Cyclopamine on KP-1N Xenografts

**Figure 1:**
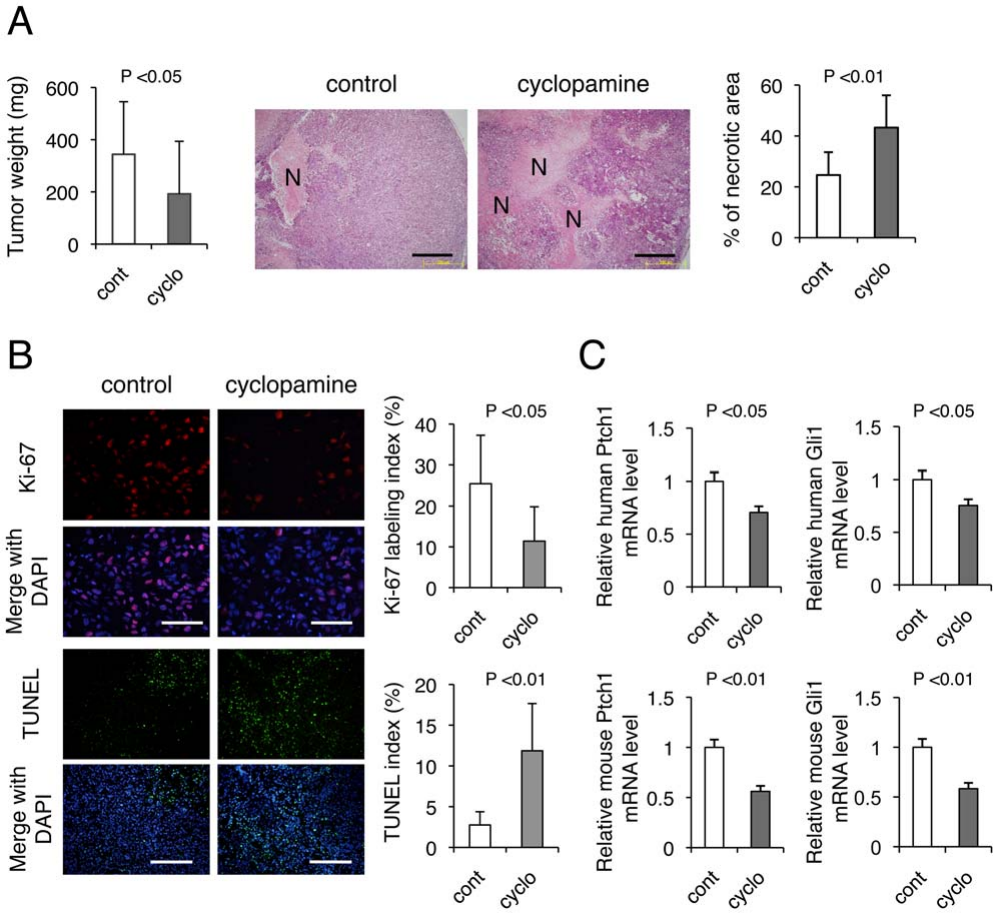
Hedgehog blockade by cyclopamine inhibits growth of PDAC xenografts *in vivo*. (A) CD-1 nude mice bearing KP-1N xenografts were treated with or without cyclopamine (50 mg/kg/day; sc) daily for 7 days (10 xenografts for each group). The results are shown as mean ± SEM tumor weight (mg). H-E stainings for xenograft sections are shown and % necrotic area (N) was measured. Scale bars; 200 µm. (B) Xenograft tissues were stained with anti-Ki-67 (Scale bars; 200 µm) and TUNEL assay (Scale bars; 500 µm) was performed. Quantification of proliferating/apoptotic is shown as percent positive cells in 5 viable fields from 10 sections. (C) RNA was extracted from xenograft tissue and mRNA levels for Ptch1 and Gli1 were quantified by TaqMan qPCR.

In order to elucidate the mechanisms by which Hh signaling might support tumor progression in a tumor cell non-autonomous fashion, we evaluated the role of Hh signaling on growth of PDAC *in vivo*. We selected a cell line, KP-1N, that exhibited high expression of Shh but was insensitive to growth inhibition by cyclopamine *in vitro* (**Figure S1, S2 and Table S2**). Treatment of KP-1N xenografts with cyclopamine daily for 7 days caused a 46-percent reduction in tumor weight as compared with vehicle control-treated tumors (**Figure 1A**). Histopathological analysis revealed that cyclopamine treated tumors had abundant areas of necrosis resulting in a 59% reduction in viable tumor weight. In line with these findings, staining for Ki-67 and TUNEL demonstrated that cyclopamine treatment reduced proliferation by 14% and increased cell death by 9% *in vivo* (**Figure 1B**); in contrast, there was no significant effect of cyclopamine on proliferation/cell death kinetics *in vitro* (**Figure S2**).

In order to determine which cells were affected by cyclopamine treatment, we measured expression of Ptch1 and Gli1 mRNA, transcriptional targets of Hh signaling, in both the xenografted human tumor cells as well as the mouse stromal compartment. To do so, we made use of species-specific probes. We observed that human Ptch1 and Gli1 in xenograft tissues were only modestly decreased by cyclopamine treatment (**Figure 1C**). Comparable, minor decreases in expression were observed *in vitro* (**Figure S3**). In comparison, mouse Ptch1 and Gli1 mRNA levels showed a markedly more pronounced downregulation, indicating that stromal cells are significant targets of Hh blockade. Collectively, these results indicate that the anti-tumor effect of cyclopamine on xenograft growth is primarily mediated through a downregulation of Hh signaling in tumor-associated stroma.

### Effects of Cyclopamine on the Tumor Vasculature

**Figure 2:**
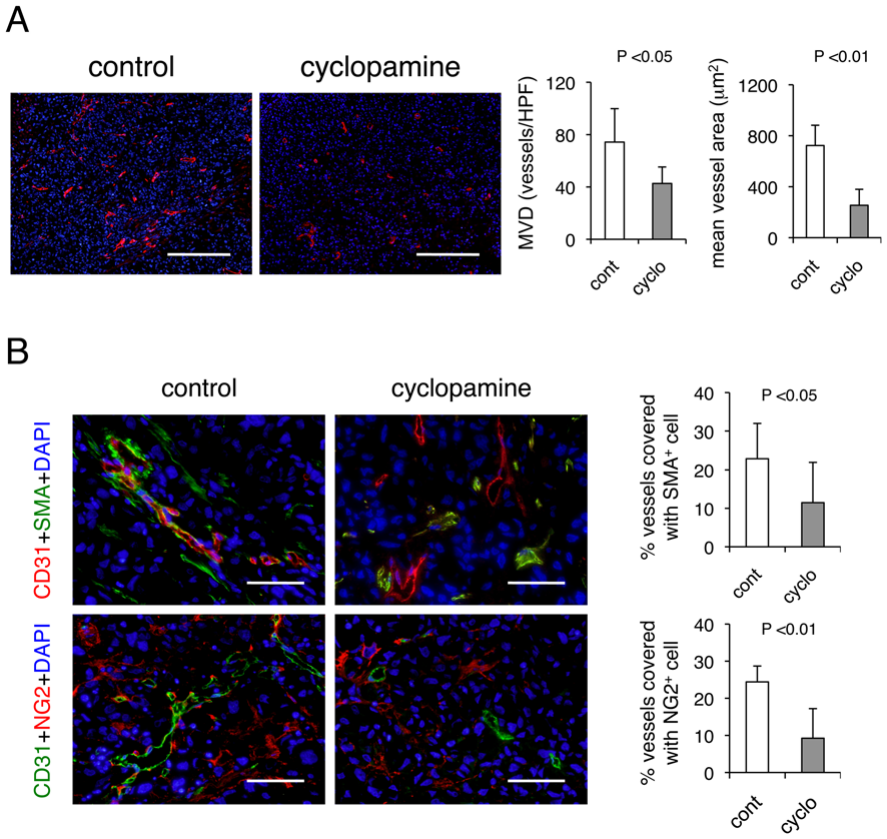
Cyclopamine inhibits the tumor angiogenesis. (A) KP-1N xenografts treated with or without cyclopamine were stained with CD31. Scale bars; 500 µm. The data are shown as mean ± SEM MVD and mean vessel area. (B) Double immunofluorescent staining with α-smooth muscle actin (SMA) with CD31 or NG2 with CD31. Scale bars; 200 µm. The number of CD31^+^ microvessels covered with α-SMA or NG2 positive pericytes is shown.

Among the stromal components in PDAC xenografts, we were particularly curious about the tumor vasculature, since we have demonstrated that BM-derived pro-angiogenic (precursor) cells are responsive to Hh ligand during neovascularization [26], [29]. Therefore, we investigated the effect of cyclopamine on the tumor vasculature in KP-1N xenografts. Immunostaining for the endothelial cell marker CD31 revealed that microvascular density was significantly reduced (42.6%) when mice were treated with cyclopamine (**Figure 2A**). The resulting tumor vasculature was narrowed and fragmented, and the reduction in mean vessel area was dramatic (64.8%). The inhibition of angiogenesis by cyclopamine was also observed in Suit-2 xenografts (**Figure S4**).

Our results suggested that blockade of Hh signaling “destabilizes” the tumor vessels. We wished to confirm this hypothesis by immunohistochemical staining for α-smooth muscle actin (SMA), NG2, and CD31. SMA and NG2 are expressed by pericytes, which are cells that closely associate with CD31-positive endothelial cells to stabilize blood vessels [30], [31]. We observed 50.0% or 62.1% fewer CD31^+^ microvessels covered with SMA^+^ or NG2^+^ pericytes in the cyclopamine treated tumors (**Figure 2B**). Thus, Hh signaling is important for maturation and/or stabilization of the tumor vasculature.

### Tumor-Derived Hh Promotes Incorporation of BM-Derived Cells into the Neovasculature

**Figure 3:**
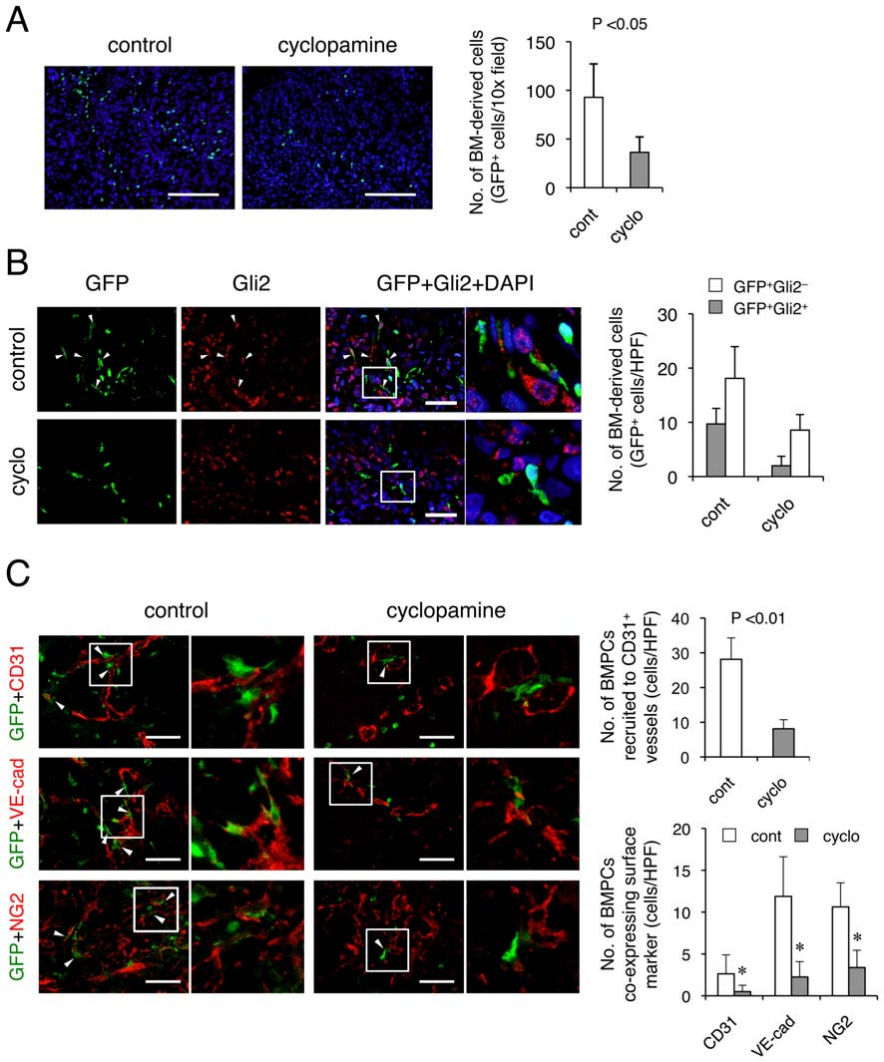
Hh promotes the recruitment of BM-derived cells into the tumor vasculature. (A-D) GFP-BMT-chimeric mice bearing KP-1N xenografts were treated with or without cyclopamine. Recruitment of BM-derived cells into the tumor was tracked by immunostaining with anti-GFP (A). The data shown as the mean ± SEM of the numbers of GFP^+^ cells. Scale Bars; 100 µm. KP-1N xenograft tissues were immunostained with Gli2 (B), CD31, VE-cadherin or NG2 (C), in combination with GFP. Scale Bars; 100 µm. Shown are mean ± SEM of GFP^+^ cells recruited to CD31^+^ tumor vessels (*upper panel*) and number of GFP^+^ cells co-localized with CD31, VE-cadherin^+^, and NG2 (*lower panel*).

Bone marrow (BM)-derived cells are thought to play a role in tumor development [32], [33]. For instance, various types of BM-derived hematopoietic cells have been observed to closely associate with the tumor neovasculature [34], [35], and indeed, a small number of BM-derived progenitor cells were demonstrated to incorporate into the lumen of a growing vasculature where they differentiate into endothelial cells in a mouse lung cancer model [36]. Various factors in the tumor microenvironment can modulate the recruitment and retention of BM-derived cells during the development of the tumor vasculature [37]. Therefore, we sought to determine whether Hh signaling contributes to this process in PDAC. In order to trace BM-derived cells, we implanted KP-1N xenografts in chimeric mice with GFP-labeled BM (GFP-bone marrow transplant model; see Methods). Cyclopamine significantly decreased the recruitment of BM-derived cells into the xenografts by 60% as compared with control tumors (**Figure 3A**).

Among Gli family of transcription factors, control of the degradation of Gli2 and the processing into its repressor form define the Hh signaling response [38]. Gli2 protein is processed and degraded in the absence of Hh ligand, whereas the presence of Hh ligand promotes Smo enrichment in primary cilia [39], resulting in nuclear translocation of full length, transcriptionally competent Gli2 protein [40]. We therefore evaluated the effect of cyclopamine on Gli2 protein via immunofluorescence analysis utilizing an antibody against full length form of Gli2 [41] (**Figure S5A**). BM-derived cells express full length Gli2 protein and cyclopamine decreased both the absolute number and the relative proportion of Gli2 positive BM-derived cells (**Figure 3B**), indicating that Hh signaling is activated in BM-derived cells in a cyclopamine-sensitive (Smo-dependent) manner. Ptch1 protein was also observed in BM-derived cells as well as in cancer cells and cyclopamine reduced the Ptch1 expression in stromal compartment **(Figure S5B**). We failed to demonstrate strong Gli1 protein expression in the xenograft tissue (data not shown).

In control tumors, GFP-positive BM-derived cells are closely associated with CD31^+^ tumor vessels but they are not incorporated to the lumen of the vasculatures (**Figure 3C**). On the other hand, a subset of BM-derived cells co-expressed VE-cadherin demonstrating that BM-derived cells are not only recruited to the peri-vascular area but also give rise directly to the tumor endothelium. The NG2 proteoglycan is expressed by nascent pericytes during the early stages of angiogenesis, and a subset of BM-derived cells in xenograft tumors co-expressed NG2. These results indicate that BM-derived cells contribute to the development of tumor vasculature not only by giving rise to specific type of cells but also by associating in different manners. Of note, cyclopamine treatment significantly decreased the integration of the GFP^+^ BM-derived pro-angiogenic cells (BMPCs) into CD31^+^, VE-cadherin^+^, or NG2^+^ tumor vasculatures. We confirmed that BMPCs were responsive to Hh since *in vitro* co-cultures with KP-1N tumor cells resulted in a cyclopamine-sensitive induction of Gli1/Ptch1 mRNA in BMPCs, but not in the mature endothelial cell line MS-1 (**Figure S6** and data not shown). Taken together, these results indicate that the Hh pathway plays a role in neovascularization by modulating the incorporation of BM-derived pro-angiogenic (precursor) cells into the tumor vessels.

### Tumor-Derived Hh Promotes Migration of BM-Derived Pro-Angiogenic Cells *In Vitro* and *In Vivo*

We speculated that the decreased numbers of BM-derived cells in the cyclopamine-treated tumors might be the result of attenuated mobilization of cells from the BM. However, the number of circulating BMPCs, as measured by labeling cultured peripheral blood MNCs with acLDL/isolectin B4 [42], was not significantly reduced in the presence of cyclopamine (**Figure S6**). This finding suggests that Hh might directly regulate recruitment, differentiation, or migration of precursors for BMPCs within the tumor microenvironment rather than affecting mobilization of the precursors from the marrow.

**Figure 4:**
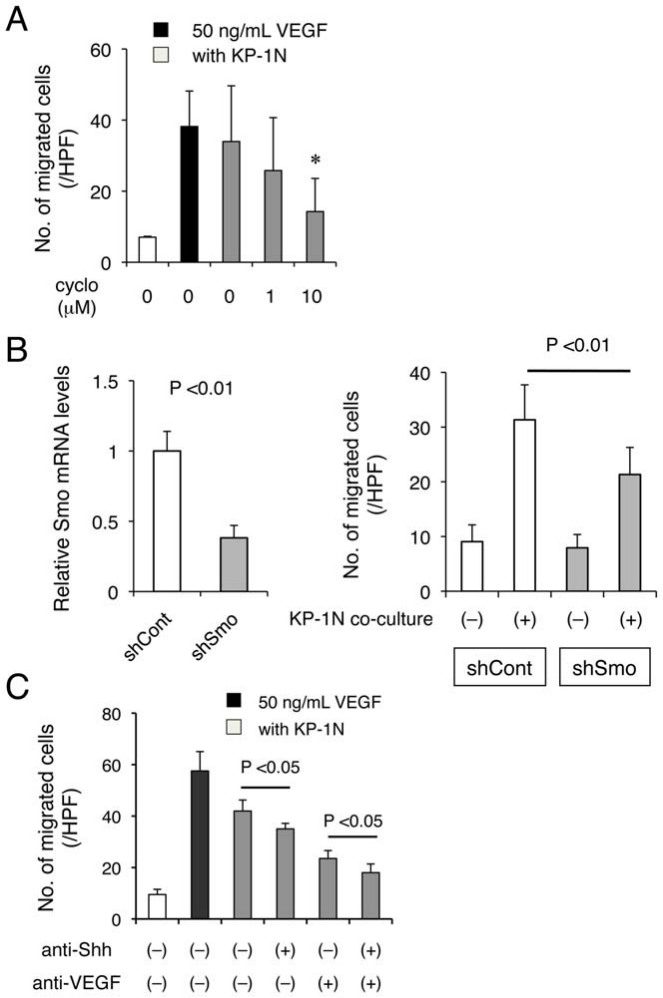
Shh derived from PDAC cells enhances migration of BM-derived pro-angiogenic cells *in vitro*. (A–B) The migration of BM-derived pre-angiogenic cells (BMPCs) was assessed. BMMNCs cultured in EGM2-MV medium for 4 days and attached cells were seeded onto transwell (5 µm pore) coated with 0.1% gelatin, and KP-1N cells were seeded in the lower well. Number of migrated cells into the bottom side of the membrane was quantified by DAPI staining after 18 h incubation. The BMPCs were pretreated with Cyclopamine (A), shRNA lentivirus for Smo (B), and anti-Shh and/or anti-VEGF neutralizing antibody (C) when seeded onto the well. For a positive control, human VEGF (50 ng/mL) was added to the lower well, and the BMPCs were cultured alone as a negative control.

Since inhibition of Hh signaling did not affect mobilization of the BMPCs, we sought to investigate the mechanisms by which Hh signaling might enhance their incorporation into the tumor vasculature. To do so, we examined the effects of Hh signaling on migration of the precursors. The migration of BMPCs through a transwell membrane *in vitro* was measured in the presence of KP-1N in the lower well. Cultured BMMNCs expressing c-Kit, CD11b and VE-cadherin were utilized as the precursors for BMPCs (**Figure S6**). The number of the BMPCs migrating to the bottom side of the membrane was significantly increased when co-cultured with KP-1N as compared with the pro-angiogenic cells without KP-1N, implying that secreted factors from the PDAC cells can enhance the migration of the precursors (**Figure 4A**). Consistent with a role for the Hh-Smo pathway, preconditioning the BMPCs with cyclopamine dramatically reduced their ability to migrate in a dose dependent manner (**Figure 4A**). To directly address the role of Smo-dependent signaling in BM-derived pro-angiogenic cells, we used the BM cells infected with an shRNA lentivirus targeting Smo (**Figure 4B**). Suppression of Smo significantly attenuated their migration to KP-1N tumor cells. The role of tumor-derived Shh in migration of the precursors was also confirmed by using a Shh neutralizing antibody (**Figure 4C**).

**Figure 5:**
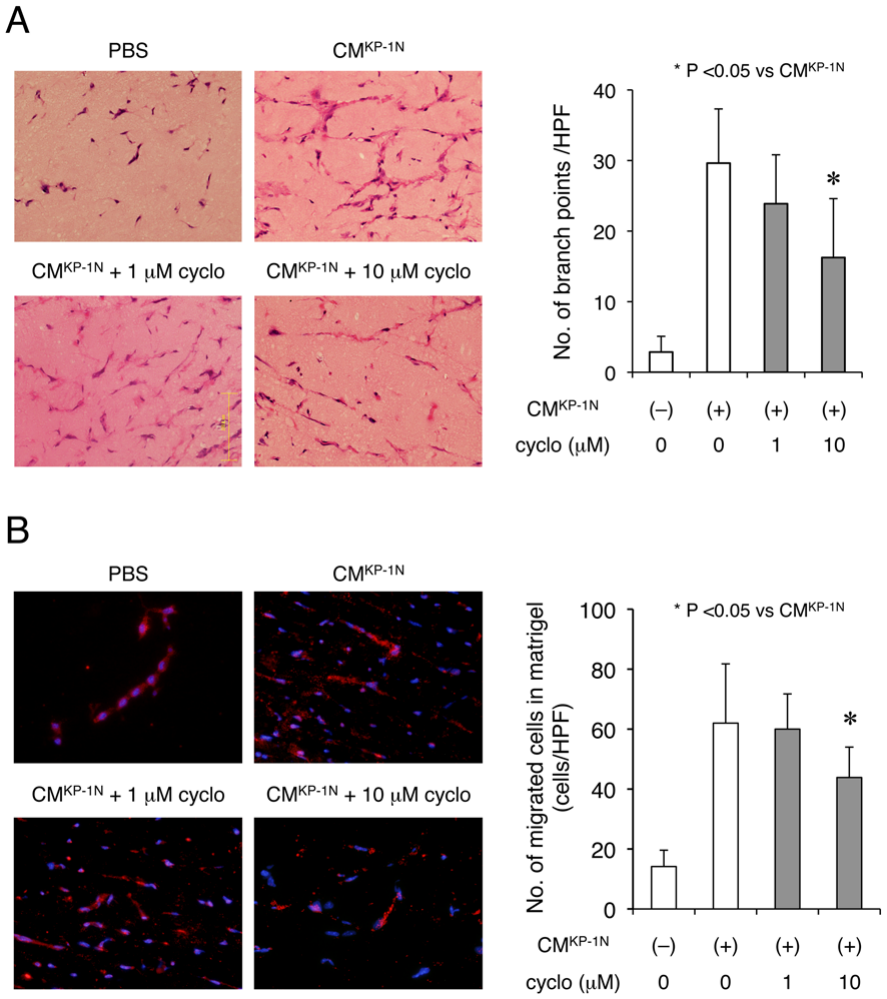
Shh derived from PDAC cells enhances recruitment of BM-derived pro-angiogenic cells *in vivo*. Matrigel plug assay utilizing Tie2/LacZ-BMT chimeric nude mice. Growth factor reduced matrigel mixed with concentrated conditioned medium from KP-1N (CM^KP-1N^) was transplanted into the chimeric mice. In experimental groups, matrigel was also mixed with cyclopamine. (A) Capillary morphogenesis within the matrigel was quantified by the number of branch points on H-E sections. (B) Matrigel was immunostained with β-gal. Shown are mean ± SEM of β-gal positive cells.

In order to elucidate the effects of Smo pathway modulation on migration of the BMPCs *in vivo*, we performed matrigel plug assays. When concentrated supernatant from KP-1N cells mixed with matrigel was implanted subcutaneously into nude mice, the number of host cells migrating into the matrigel was significantly increased, and the cells formed a cord-like structure, mimicking capillary morphogenesis *in vivo* (**Figure 5A**). In order to distinguish BM-derived cells from other stromal cells, the experiment was repeated by utilizing other chimeric mice that underwent BMT. Since we observed significant Tie2 mRNA expression in the BM-derived pro-angiogenic cells utilized in *in vitro* assays (data not shown), Tie2/LacZ mice were used as donor for the BMT [42]. A large fraction of the cells within matrigel originated from BM as reflected by positive immunostaining for β-gal (**Figure 5B**). The migration of host cells into matrigel plugs was attenuated significantly when cyclopamine was added to the matrigel. A similar result was also obtained using an anti-Shh antibody (data not shown).

Collectively, our results demonstrate that inhibition of tumor-derived Hh signaling leads to inhibition of tumor angiogenesis and destabilization of vasculature with a concomitant decrease in recruitment of BMPCs into the tumor stroma.

### Regulation of Pro- and Anti-Angiogenic Factors by Hh Signaling

To identify the molecular basis of the vascular disruption by Hh blockade, we quantified the mRNA expression levels of pro- and anti-angiogenic factors in PDAC xenografts using species-specific primer/probe sets for qPCR. Specifically, we were curious to know the levels of VEGF and SDF-1, which are considered pro-angiogenic factors due to their demonstrated effects on the recruitment and retention of the pro-angiogenic precursor cells at sites of tumor formation [34], [43].

We found that human VEGF and SDF-1 mRNA expression were decreased by 31% and 33%, respectively, in the cyclopamine-treated tumors (**Figure S7**). These results are in contrast to the modest downregulation of VEGF and SDF-1 mRNA in cyclopamine-treated KP-1N cells *in vitro*, either in normoxic or hypoxic conditions (**Figure S8**). Likewise, HIF-1α stability was not affected under hypoxic conditions *in vitro*. Moreover, blocking Shh by neutralizing antibody did not cause a reduction in VEGF mRNA in KP-1N cells, suggesting that autocrine Shh signaling does not impart an angiogenic phenotype directly on the KP-1N (**Figure S8**). Instead, these collective results suggest that Hh signaling impinges upon the stroma and that stromal-derived factors might contribute to the expression of VEGF and SDF-1 in the tumor cells *in vivo*.

**Figure 6:**
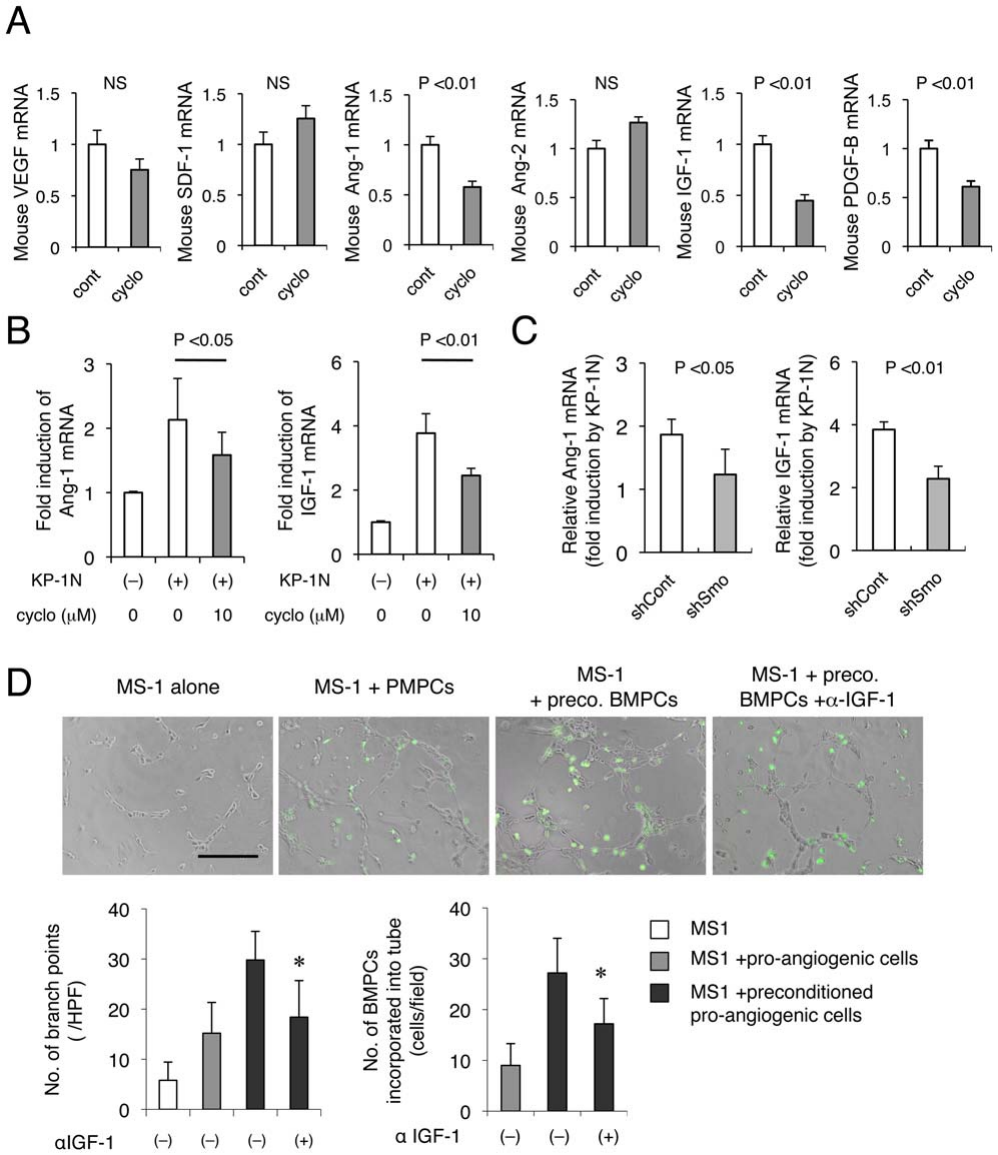
Shh derived from PDAC cells induces in Ang-1 and IGF-1 in BM-derived pro-angiogenic cells. (A) mRNA expressions of murine pro-/anti-angiogenic factors in xenografts were quantified by qPCR. (B, C) Co-culture assay was performed utilizing trenswell. BM-derived pro-angiogenic cells (BMPCs) were infected with a lentivirus expressing control shRNA or Smo shRNA and co-culture with KP-1N cells seeded onto transwell (0.4 µm). RNA was harvested from the BMPCs 12 h after for qPCR (normalized to the cells without KP-1N). (D) Tube formation assay was performed by culturing MS-1 on matrigel in the presence or absence of GFP-labeled BMPCs. Capillary morphogenesis was observed after 8 h incubation with or without anti-IGF-1 neutralizing antibody. Scale bar, 500 µm. Number of branch points and incorporation of the BMPCs into tube was quantified.

It has been demonstrated that cancer associated fibroblasts secrete SDF-1 to recruit bone marrow-derived endothelial precursor cells into the tumor stroma in order to mediate angiogenesis [44]. Therefore, we next quantified the expression of these pro-/anti-angiogenic factors by the murine tumor stroma. Mouse VEGF and SDF-1 levels were not affected by cyclopamine *in vivo* (**Figure 6A**), indicating that Hh is not involved in the modulation of these pro-angiogenic factors by tumor stroma.

We next examined the expression of factors that modulate vascular stability. In the tumors of cyclopamine-treated mice, we noted a marked reduction in murine Ang-1, IGF-1, and PDGF-B mRNA, factors known to stabilize the neovasculature. Ang-1 plays a crucial role during angiogenesis to stabilize neovessels [30], and its cognate Tie2 receptor is expressed in ECs, pro-angiogenic monocytes as well as pericyte precursors of mesenchymal origin [45]. The reduction of Ang-1 was coupled with 86.3% upregulation of Ang-2, a natural antagonist for Tie2, that leads to dissociation of the mural cell coating to initiate angiogenesis [46] (**Figure S9**). In an effort to understand the regulation of these factors by Hh signaling, we compared their mRNA levels in BMPCs that were co-cultured with KP-1N cells. Ang-1 and IGF-1 but not PEDF-B mRNA was upregulated by more than 2-fold in a cyclopamine-sensitive manner (**Figure 6B**), and simultaneous downregulation of Ang-2 mRNA in the precursors was observed. The altered expression of Ang-1 and Ang-2 was canceled by cyclopamine, and the effect on Ang-1/Ang-2 ratio was dramatic (54.3% reduction; **Figure S9**). Consistent results were observed when we targeted Smo in the BM-derived precursors by lentiviral shRNA, confirming that the alteration of Ang-1 gene expression by cyclopamine was due to blockade of Smo-dependent pathway (**Figure 6C**). Thus, Hh-dependent Ang-1/Tie2 signaling appears to be one of the potential mechanisms by which BMPCs stabilize neovessels.

### IGF-1 Is a Target of Hh Signaling in BM-Derived Pro-Angiogenic Cells

The Reduction in IGF-1 mRNA that we observed in cyclopamine treated xenografts (**Figure 6A**) was also recapitulated in the co-culture assays; i.e., the strong induction of IGF-1 in BMPCs by KP-1N-derived factors was blocked significantly either by cyclopamine or shRNA against Smo (**Figure 6B, C**). To verify the role of IGF-1 during neovascularization, a tube formation assay was performed using MS-1 (mouse mature EC line) with GFP-labeled BM-derived pro-angiogenic precursors (**Figure 6D**). Capillary morphogenesis by MS-1 was induced by the BMPCs, and the effect was enhanced when they were preconditioned in the presence of KP-1N cells. Strikingly, the effect on capillary formation was significantly reduced upon treatment with an anti-IGF-1 neutralizing antibody. Thus, IGF-1 is an important target of Hh signaling in BM-derived cells, and the downregulation of IGF-1 in tumor xenografts by cyclopamine treatment could contribute to the disruption of angiogenesis.

## Discussion

In the current study, we identified the BM-derived pro-angiogenic cells (BMPCs) as one of the paracrine targets of Hh, which plays a critical role to promote tumor angiogenesis and tumor progression. The destabilized tumor vasculature that we observed upon cyclopamine treatment first lead us to speculate that we might observe a reduction in stromal Ang-1 and IGF-1, factors known to be regulated by Hh pathway [47], [48].

Ang-1 develops and stabilizes the primitive vasculature under Hh signaling in lung branching morphogenesis [49] and Shh increased the mRNA levels of Ang-1 in fibroblasts, but decreased Ang-2 [47], [50]. Our co-culture experiment demonstrated Ang-1 mRNA in BMPCs was upregulated by soluble factors from KP-1N in a Smo-dependent manner, suggesting a role of the precursors as reservoir of Ang-1 in the tumor microenvironment to stabilize Tie2-expressing ECs as well as subsets of pro-angiogenic cells. Indeed, a certain fraction of BM-derived cells also express Tie-2 receptor [51], supporting an alternative autocrine or paracrine effect on the their angiogenic activity by the surrounding stroma via Ang-1 ligand.

There are various cellular sources of IGF-1 in the tumor stroma, and the reduction in stroma-derived IGF-1 that we observed may explain the growth inhibition of PDAC cells *in vivo* when Hh signaling was blocked [27]. IGF-1 can also enhance the activity of BM-derived cells to promote neovascularization through PI3K/Akt signaling [52]. In the current study, the enhancement of capillary morphogenesis of MS-1 cells by the BMPCs stimulated with PDAC cells was clearly attenuated by anti-IGF-1 (Figure 5D). Thus, IGF-1 appears to be one of the crucial targets of Hh signaling in the BMPCs and mediates its effect on neovascularization. There was a significant reduction in PDGF-B expression in the tumor stroma *in vivo* as well as in BMPCs *in vitro*; however, our co-culture assay of BMPCs with KP-1N did not demonstrate upregulation of PDGF-B mRNA, suggesting that cancer-derived Hh ligand is unlikely to play a major role in the induction of PDGF-B. However, the downregulation of PDGF-B by cyclopamine may partially account for the decreased recruitment of pericytes into newly formed vascular network in xenografts.

The molecular mechanisms that we uncovered here clearly demonstrate that Hh signaling plays a paracrine role in tumor progression. It was recently reported that transcriptional induction of Gli can be maintained independent of Smo [24], and the non-canonical regulation of Gli-target genes is mediated in part through TGF-β and Kras signaling. Indeed, most PDAC cells, including KP-1N, express oncogenic Kras and it has been demonstrated that TGF-β-Smad pathway is activated in KP-1N cells [53]. These pathways might account for the modest reduction of Gli1 transcription by cyclopamine that we observed. It is also possible that the *in vivo* growth inhibition by Hh blockade that we observed might be mediated through an effect on self-renewal property of rare PDAC initiating cells [54]. However, the significant change in proliferation/death kinetics that we observed in a large proportion of cancer cells in the xenografts treated with cyclopaminewas clearly accompanied by the inhibition of angiogenesis.

Cyclopamine attenuated not only the recruitment of BM-derived cells to the tumor but also their incorporation into the tumor vasculature, supporting an involvement of Hh in the tumor-associated angiogenesis. Our data do not support the notion that autocrine signaling of Hh in cancer cells contributes to tumor angiogenesis, and rather suggest that Hh acts exclusively on the stroma. Shh has been shown to directly control the activity of BMPCs cells by stimulating their proliferation, migration and inducing angiogenic factors to promote vascular remodeling [26], [29], [55]. We demonstrated that factors secreted by PDAC cells can enhance migration of the precursor cells at least in part in a Smo-dependent manner. There was an additional effect of Hh signaling on EC assembly through an activation of the BMPCs together with sprouting mature ECs. We have previously shown that Shh can enhance the activity of the precursors to promote capillary morphogenesis by HUVEC [26]. This is probably mediated through factors “imported” by the pro-angiogenic precursors that can prime neovascularization. BM-derived cells can differentiate into various cell types in the tumor microenvironment and the anti-tumor effect of Hh blockade may be mediated through multiple players in the cancer stroma [33]. Mesenchymal stem cells and immune cells are alternative targets of Hh [56], [57]. In addition, fibroblasts in PDAC desmoplasia could be important Hh reactive cells [25]. Bailey *et al.* demonstrated that fibroblasts in human PDAC were positive for Gli1, and Shh promoted the proliferation of pancreatic stellate cells and induced their differentiation into myofibroblasts [28]. A similar conclusion was reached based on experiments showing that wild type, but not Smo knockout, mouse embryonic fibroblasts promote the growth of PDAC xenografts [27]. More recent study by Olive *et al.* indicated that IPI-926, a semisynthetic derivative of cyclopamine, can inhibit proliferation of SMA-positive stromal myofibroblasts, resulting in a depletion of desmoplastic PDAC stroma [22]. Although they described a transient ‘enhancement’ of tumor angiogenesis by Smo inhibition, the reduction in SMA^+^ myofibroblasts may limit the maturation of neovasculature through a depletion of vascular pericytes, which in turn consequently ‘destabilize’ tumor-associated neovessels as we and others [58] observed. Further studies are required to monitor the tumor vessels during longer periods of Hh signaling inhibition in types of tumor with diverse histological architectures.

Our proposed model for Hh-mediated tumor angiogenesis is illustrated in **Figure S10**. The paracrine effect of Hh emerges at late stages of tumorigenesis and regulates neovascularization by acting through BM-derived cells. Hh ligand stimulates migration/interaction of the precursors to neovessels, which in turn act as a reservoir of cytokines to stabilize/maintain the tumor vasculature. The inhibition of angiogenesis by targeting Smo is consistent with earlier studies demonstrating a pro-angiogenic role for Hh on ischemic tissues [29], [47], embryonic vasculogenesis [59], [60], as well as in tumor [58]. Hh could also contribute to the activation of stromal myofibroblasts (potentially derived from the BM) that promote tumorigenesis through supporting angiogenesis or other mechanisms [22]. However, so far the possibility that Hh signaling may affect on pre-existing vascular cells as well still exists. In addition, the potential early cell-autonomous role needs further investigation since it is not easily reconciled with the dispensability of epithelial Smo expression for PanIN initiation and progression [24].

Together with recent studies [24], [27], [28], it is becoming clear that a paracrine effect of Hh is crucial for tumorigenesis. Activation of the Hh pathway is important for the stromal cells, rather than aberrant proliferation of cancer cells. We have identified a novel aspect of Hh as a late mediator to support the tumor microenvironment, which promotes the development of the tumor vasculature in a paracrine manner, and this is mediated by a significant effect on BM-derived pro-angiogenic cells.

## Supporting Information

Methods S1 Supplementary methods.
(0.12 MB DOC)

Figure S1 Expression of Hh signaling components. Quantitative RT-PCR profiling of Hh pathway genes was assessed by Taqman assay.
(0.73 MB TIF)

Figure S2 Effects of cyclopamine on human PDAC cell proliferation/death *in vitro*. (A) Cell proliferation assay was performed in the presence of cyclopamine. (B) KP-1N and Suit-2 cells were cultured with cyclopamine for 7 days. The culture medium containing fresh cyclopamine was changed every 2 days. (C) Annexin V flow cytometry analysis. KP-1N cells were treated with 1–10 µM cyclopamine for 24 hours. The early apoptotic fraction was quantified as FITC-Annexin V+/PI- fraction (indicated by red frame).
(5.75 MB TIF)

Figure S3 Reduction of Gli1/Gli2 mRNA levels by cyclopamine in pancreatic cancer cells *in vitro*. KP-1N and Suit-2 cells were treated with 1–10 µM of cyclopamine (cyclo) for 8 hours and Gli1 and Gli2 mRNA expression was quantified.
(0.51 MB TIF)

Figure S4 Cyclopamine inhibits growth and tumor angiogenesis of Suit-2 xenogtafts. CD-1 nude mice bearing Suit-2 xenografts were treated with or without cyclopamine (50 mg/kg/day, dissolved in PBS containing 10% 2-hydroxylpropyl-h-cyclodextrin at a concentration of 2.5 mg/mL) administrated by oral daily for 7 days (6 xenografts for each group). The results are shown as mean ± SEM tumor weight (mg). Sections were stained with anti-CD31. Scale bars; 500 µm. The data are shown as mean ± SEM MVD and mean vessel area.
(0.88 MB TIF)

Figure S5 Downregulation of full length Gli2 and Ptch1 expression in stroma by Hedgehog blockade. (A) KP-1N xenografts treated with or without cyclopamine were stained with anti-Gli2 antibody (Abcam, ab26056 and SantaCruz, G-20). (B) KP-1N xenograft tissues on GFP-BMT-chimeric mice were immunostained with Ptch1 in combination with GFP. The results shown are mean number of GFP+ BM-derived cells positive and negative for Ptch1. Scale bars; 100 µm.
(9.54 MB TIF)

Figure S6 Characterization of cultured BM-derived pro-angiogenic cells. BMMNCs cultured with EGM2-MV medium (10% FBS) in vitronectin-coated dish were utilized as BM-derived pro-angiogenic cells (BMPCs) for *in vitro* experiments. (A) After 4–7 days culture, spindle shaped attached cells were incubated with acetylated LDL (acLDL; DiI labeled) and labeled with BS1-lectin (FITC-conjugated). Flow cytometric analysis of attached cells on day 7. Data show the percentage of positive cells for progenitor and endothelial markers. (B) BMPCs were seeded in the lower well and co-cultured with KP-1N cells in the upper well in the presence or absence of 10 µM cyclopamine. RNA was harvested from the BMPCs 12 h after for qPCR analysis (normalized to the cells without KP-1N). (C) Circulating BMPCs in KP-1N xenograft-bearing mice are quantified. MNCs from 500 µL blood were cultured with EGM2-MV medium for 4 days and labeled with DiI-acLDL/FITC-isolectin B4. The results shown as the mean ± SEM number of circulating BMPCs (double positive cells/field).
(1.98 MB TIF)

Figure S7 Cyclopamione downregulate human VEGF and SDF-1 in KP-1N xenografts. mRNA expressions of human pro-/anti-angiogenic factors in xenografts were quantified by qPCR.
(0.41 MB TIF)

Figure S8 Hedgehog blockade does not reduce VEGF and SDF-1 mRNA expression in KP-1N cells *in vitro*. (A) KP-1N were treated with 1–10 µM cyclopamine for 1 h and then cultured either in normoxic (20% O2) or hypoxic (1% O2) conditions. Protein lysates were harvested after 8 h incubation to detect HIF-1α protein by western blotting. Immunoblot analysis for anti-HIF-1α (clone 54, 1∶250, BD) and β-actin (1∶5000, Sigma) was performed. (B, C) Total RNA was extracted after 8 h incubation with 1–10 µM cyclopamine or 1–10 µg/mL anti-Shh (MAB4641; R&D systems) under normoxic or hypoxic conditions to quantify VEGF and SDF-1 mRNA by qPCR.
(1.10 MB TIF)

Figure S9 VEGF, Ang-2 and PDGF-B expression in BM-derived pro-angiogenic cells was downregulated by cyclopamine. (A and B) Mouse BM-derived pro-angiogenic cells (BMPCs) were co-cultured with KP-1N utilizing transwell (0.4 µm pore). BMPCs were seeded on lower well and KP-1N cells on upper well. mRNA levels for mouse VEGF (A) Ang-1/Ang-2 (B), and PDGF-B (C) in the BMPCs were quantified by qPCR after 12 h co-culture with or without 10 µM cyclopamine (normalized to the BMPCs without KP-1N).
(0.52 MB TIF)

Figure S10 Proposed model for Hh-mediated angiogenesis in PDAC. Shh is an early mediator for pancreatic tumorigenesis, but it may not be sufficient for proliferation of cancer cells at late stages. The paracrine effect of Hh emerges during tumorigenesis by acting through BM-derived cells including pro-agiogenic (precursor) cells and contributing to the development and maintenance of the tumor vasculature. Hh could also contribute to the activation of stromal fibroblasts (potentially derived from the BM) that potentially promote tumorigenesis through supporting angiogenesis or other mechanisms.
(0.74 MB TIF)

Table S1 (0.05 MB DOC)

Table S2 (0.04 MB DOC)

## References

[1] Matsuno S, Egawa S, Fukuyama S, Motoi F, Sunamura M (2004). Pancreatic Cancer Registry in Japan: 20 years of experience.. Pancreas.

[2] Jemal A, Siegel R, Ward E, Hao Y, Xu J (2008). Cancer statistics, 2008.. CA Cancer J Clin.

[3] Burris HA, Moore MJ, Andersen J, Green MR, Rothenberg ML (1997). Improvements in survival and clinical benefit with gemcitabine as first-line therapy for patients with advanced pancreas cancer: a randomized trial.. J Clin Oncol.

[4] Moore MJ, Goldstein D, Hamm J, Figer A, Hecht JR (2007). Erlotinib plus gemcitabine compared with gemcitabine alone in patients with advanced pancreatic cancer: a phase III trial of the National Cancer Institute of Canada Clinical Trials Group.. J Clin Oncol.

[5] Rinehart J, Adjei AA, Lorusso PM, Waterhouse D, Hecht JR (2004). Multicenter phase II study of the oral MEK inhibitor, CI-1040, in patients with advanced non-small-cell lung, breast, colon, and pancreatic cancer.. J Clin Oncol.

[6] Saif MW (2007). Pancreatic cancer: is this bleak landscape finally changing? Highlights from the ‘43rd ASCO Annual Meeting’. Chicago, IL, USA. June 1-5, 2007.. JOP.

[7] Thayer SP, di Magliano MP, Heiser PW, Nielsen CM, Roberts DJ (2003). Hedgehog is an early and late mediator of pancreatic cancer tumorigenesis.. Nature.

[8] Morton JP, Mongeau ME, Klimstra DS, Morris JP, Lee YC (2007). Sonic hedgehog acts at multiple stages during pancreatic tumorigenesis.. Proc Natl Acad Sci U S A.

[9] Kawahira H, Scheel DW, Smith SB, German MS, Hebrok M (2005). Hedgehog signaling regulates expansion of pancreatic epithelial cells.. Dev Biol.

[10] Ohuchida K, Mizumoto K, Fujita H, Yamaguchi H, Konomi H (2006). Sonic hedgehog is an early developmental marker of intraductal papillary mucinous neoplasms: clinical implications of mRNA levels in pancreatic juice.. J Pathol.

[11] Roberts DJ, Johnson RL, Burke AC, Nelson CE, Morgan BA (1995). Sonic hedgehog is an endodermal signal inducing Bmp-4 and Hox genes during induction and regionalization of the chick hindgut.. Development.

[12] Schuller U, Zhao Q, Godinho SA, Heine VM, Medema RH (2007). Forkhead transcription factor FoxM1 regulates mitotic entry and prevents spindle defects in cerebellar granule neuron precursors.. Mol Cell Biol.

[13] Ingram WJ, Wicking CA, Grimmond SM, Forrest AR, Wainwright BJ (2002). Novel genes regulated by Sonic Hedgehog in pluripotent mesenchymal cells.. Oncogene.

[14] Elia D, Madhala D, Ardon E, Reshef R, Halevy O (2007). Sonic hedgehog promotes proliferation and differentiation of adult muscle cells: Involvement of MAPK/ERK and PI3K/Akt pathways.. Biochim Biophys Acta.

[15] Hoseong Yang S, Andl T, Grachtchouk V, Wang A, Liu J (2008). Pathological responses to oncogenic Hedgehog signaling in skin are dependent on canonical Wnt/beta-catenin signaling.. Nat Genet.

[16] Zhu G, Mehler MF, Zhao J, Yu Yung S, Kessler JA (1999). Sonic hedgehog and BMP2 exert opposing actions on proliferation and differentiation of embryonic neural progenitor cells.. Dev Biol.

[17] Huangfu D, Anderson KV (2006). Signaling from Smo to Ci/Gli: conservation and divergence of Hedgehog pathways from Drosophila to vertebrates.. Development.

[18] Incardona JP, Gaffield W, Kapur RP, Roelink H (1998). The teratogenic Veratrum alkaloid cyclopamine inhibits sonic hedgehog signal transduction.. Development.

[19] Berman DM, Karhadkar SS, Hallahan AR, Pritchard JI, Eberhart CG (2002). Medulloblastoma growth inhibition by hedgehog pathway blockade.. Science.

[20] Watkins DN, Berman DM, Burkholder SG, Wang B, Beachy PA (2003). Hedgehog signalling within airway epithelial progenitors and in small-cell lung cancer.. Nature.

[21] Feldmann G, Dhara S, Fendrich V, Bedja D, Beaty R (2007). Blockade of hedgehog signaling inhibits pancreatic cancer invasion and metastases: a new paradigm for combination therapy in solid cancers.. Cancer Res.

[22] Olive KP, Jacobetz MA, Davidson CJ, Gopinathan A, McIntyre D (2009). Inhibition of Hedgehog Signaling Enhances Delivery of Chemotherapy in a Mouse Model of Pancreatic Cancer.. Science.

[23] Feldmann G, Fendrich V, McGovern K, Bedja D, Bisht S (2008). An orally bioavailable small-molecule inhibitor of Hedgehog signaling inhibits tumor initiation and metastasis in pancreatic cancer.. Mol Cancer Ther.

[24] Nolan-Stevaux O, Lau J, Truitt ML, Chu GC, Hebrok M (2009). GLI1 is regulated through Smoothened-independent mechanisms in neoplastic pancreatic ducts and mediates PDAC cell survival and transformation.. Genes Dev.

[25] Tian H, Callahan CA, DuPree KJ, Darbonne WC, Ahn CP (2009). Hedgehog signaling is restricted to the stromal compartment during pancreatic carcinogenesis.. Proc Natl Acad Sci U S A.

[26] Yamazaki M, Nakamura K, Mizukami Y, Ii M, Sasajima J (2008). Sonic hedgehog derived from human pancreatic cancer cells augments angiogenic function of endothelial progenitor cells.. Cancer Sci.

[27] Yauch RL, Gould SE, Scales SJ, Tang T, Tian H (2008). A paracrine requirement for hedgehog signalling in cancer.. Nature.

[28] Bailey JM, Swanson BJ, Hamada T, Eggers JP, Singh PK (2008). Sonic hedgehog promotes desmoplasia in pancreatic cancer.. Clin Cancer Res.

[29] Asai J, Takenaka H, Kusano KF, Ii M, Luedemann C (2006). Topical sonic hedgehog gene therapy accelerates wound healing in diabetes by enhancing endothelial progenitor cell-mediated microvascular remodeling.. Circulation.

[30] Yamada Y, Takakura N (2006). Physiological pathway of differentiation of hematopoietic stem cell population into mural cells.. J Exp Med.

[31] McCarty MF, Somcio RJ, Stoeltzing O, Wey J, Fan F (2007). Overexpression of PDGF-BB decreases colorectal and pancreatic cancer growth by increasing tumor pericyte content.. J Clin Invest.

[32] Kerbel RS (2008). Tumor angiogenesis.. N Engl J Med.

[33] Joyce JA, Pollard JW (2009). Microenvironmental regulation of metastasis.. Nat Rev Cancer.

[34] Grunewald M, Avraham I, Dor Y, Bachar-Lustig E, Itin A (2006). VEGF-induced adult neovascularization: recruitment, retention, and role of accessory cells.. Cell.

[35] Conejo-Garcia JR, Buckanovich RJ, Benencia F, Courreges MC, Rubin SC (2005). Vascular leukocytes contribute to tumor vascularization.. Blood.

[36] Gao D, Nolan DJ, Mellick AS, Bambino K, McDonnell K (2008). Endothelial progenitor cells control the angiogenic switch in mouse lung metastasis.. Science.

[37] Kopp HG, Ramos CA, Rafii S (2006). Contribution of endothelial progenitors and proangiogenic hematopoietic cells to vascularization of tumor and ischemic tissue.. Curr Opin Hematol.

[38] Pan Y, Bai CB, Joyner AL, Wang B (2006). Sonic hedgehog signaling regulates Gli2 transcriptional activity by suppressing its processing and degradation.. Mol Cell Biol.

[39] Corbit KC, Aanstad P, Singla V, Norman AR, Stainier DY (2005). Vertebrate Smoothened functions at the primary cilium.. Nature.

[40] Haycraft CJ, Banizs B, Aydin-Son Y, Zhang Q, Michaud EJ (2005). Gli2 and Gli3 localize to cilia and require the intraflagellar transport protein polaris for processing and function.. PLoS Genet.

[41] Nielsen SK, Mollgard K, Clement CA, Veland IR, Awan A (2008). Characterization of primary cilia and Hedgehog signaling during development of the human pancreas and in human pancreatic duct cancer cell lines.. Dev Dyn.

[42] Ii M, Nishimura H, Iwakura A, Wecker A, Eaton E (2005). Endothelial progenitor cells are rapidly recruited to myocardium and mediate protective effect of ischemic preconditioning via “imported” nitric oxide synthase activity.. Circulation.

[43] Yamaguchi J, Kusano KF, Masuo O, Kawamoto A, Silver M (2003). Stromal cell-derived factor-1 effects on ex vivo expanded endothelial progenitor cell recruitment for ischemic neovascularization.. Circulation.

[44] Orimo A, Gupta PB, Sgroi DC, Arenzana-Seisdedos F, Delaunay T (2005). Stromal fibroblasts present in invasive human breast carcinomas promote tumor growth and angiogenesis through elevated SDF-1/CXCL12 secretion.. Cell.

[45] De Palma M, Venneri MA, Galli R, Sergi Sergi L, Politi LS (2005). Tie2 identifies a hematopoietic lineage of proangiogenic monocytes required for tumor vessel formation and a mesenchymal population of pericyte progenitors.. Cancer Cell.

[46] Maisonpierre PC, Suri C, Jones PF, Bartunkova S, Wiegand SJ (1997). Angiopoietin-2, a natural antagonist for Tie2 that disrupts in vivo angiogenesis.. Science.

[47] Pola R, Ling LE, Silver M, Corbley MJ, Kearney M (2001). The morphogen Sonic hedgehog is an indirect angiogenic agent upregulating two families of angiogenic growth factors.. Nat Med.

[48] Rao G, Pedone CA, Del Valle L, Reiss K, Holland EC (2004). Sonic hedgehog and insulin-like growth factor signaling synergize to induce medulloblastoma formation from nestin-expressing neural progenitors in mice.. Oncogene.

[49] van Tuyl M, Groenman F, Wang J, Kuliszewski M, Liu J (2007). Angiogenic factors stimulate tubular branching morphogenesis of sonic hedgehog-deficient lungs.. Dev Biol.

[50] Lee SW, Moskowitz MA, Sims JR (2007). Sonic hedgehog inversely regulates the expression of angiopoietin-1 and angiopoietin-2 in fibroblasts.. Int J Mol Med.

[51] Rafii S, Lyden D (2003). Therapeutic stem and progenitor cell transplantation for organ vascularization and regeneration.. Nat Med.

[52] Thum T, Hoeber S, Froese S, Klink I, Stichtenoth DO (2007). Age-dependent impairment of endothelial progenitor cells is corrected by growth-hormone-mediated increase of insulin-like growth-factor-1.. Circ Res.

[53] Ijichi H, Ikenoue T, Kato N, Mitsuno Y, Togo G (2001). Systematic analysis of the TGF-beta-Smad signaling pathway in gastrointestinal cancer cells.. Biochem Biophys Res Commun.

[54] Clement V, Sanchez P, de Tribolet N, Radovanovic I, Ruiz i Altaba A (2007). HEDGEHOG-GLI1 signaling regulates human glioma growth, cancer stem cell self-renewal, and tumorigenicity.. Curr Biol.

[55] Kusano KF, Pola R, Murayama T, Curry C, Kawamoto A (2005). Sonic hedgehog myocardial gene therapy: tissue repair through transient reconstitution of embryonic signaling.. Nat Med.

[56] Lin N, Tang Z, Deng M, Zhong Y, Lin J (2008). Hedgehog-mediated paracrine interaction between hepatic stellate cells and marrow-derived mesenchymal stem cells.. Biochem Biophys Res Commun.

[57] Fontaine C, Cousin W, Plaisant M, Dani C, Peraldi P (2008). Hedgehog signaling alters adipocyte maturation of human mesenchymal stem cells.. Stem Cells.

[58] Guimaraes AR, Rakhlin E, Weissleder R, Thayer SP (2008). Magnetic resonance imaging monitors physiological changes with antihedgehog therapy in pancreatic adenocarcinoma xenograft model.. Pancreas.

[59] Rowitch DH, St-Jacques B, Lee SM, Flax JD, Snyder EY (1999). Sonic hedgehog regulates proliferation and inhibits differentiation of CNS precursor cells.. J Neurosci.

[60] Pepicelli CV, Lewis PM, McMahon AP (1998). Sonic hedgehog regulates branching morphogenesis in the mammalian lung.. Curr Biol.

