# Supplementary material for: Hedgehog Promotes Neovascularization in Pancreatic Cancers by Regulating Ang-1 and IGF-1 Expression in Bone-Marrow Derived Pro-Angiogenic Cells
Source: PLoS One. 2010 Jan 21;5(1):e8824. doi: 10.1371/journal.pone.0008824 (PMC2809097; doi:10.1371/journal.pone.0008824)
Supplement: Methods S1 — Supplementary methods. (0.12 MB DOC) [file pone.0008824.s001.doc]

**Supplementary Methods**

***Cell Culture***

Six human pancreatic adenocarcinoma cell lines, KP-1N, Suit-2 (both from Health Science Research Resources Bank, Osaka, Japan), PK1, MIAPaca2 (both from Cell Resource Center for Biochemical Research, Tohoku University, Sendai, Japan), Panc1, BxPC3 (both from ATCC), and mice endothelial cell line MS-1 (from ATCC) were used in this study.

Cell viability was assessed by WST assay (Quick Cell Proliferation Assay Kit, Biovision) [1]. Briefly, pancreatic cancer cells were plated in 96-well plates (5 x10^3^ cells/well) and grown up to 72 hours with or without cyclopamine. The number of cells was quantified using a microtiter plate reader at 450 nm according to the manufacturer’s instructions.

***Xenograft tumor model***

Administration of cyclopamine administration *in vivo* was performed as described previously [2]. Briefly, 0.1 mL RPMI and Matrigel (Becton Dickinson) (2:1) containing 5 x10^6^ KP-1N cells were injected subcutaneously into female CD-1 nude mice and tumors were grown to minimum volume of 125 mm^3^. Animals were then treated with 0.1 mL vehicle (triolein:ethanol 4:1 vol./vol.) or with a cyclopamine (50 mg/kg/day, Biomol) suspension injected subcutaneously into the animal daily for 7 days for KP-1N xenografts. Additional cyclopamine treatment was performed using Suit-2 xenograft. Cyclopamine was orally administrated as described previously [3]. Briefly, PBS or cyclopamine (50 mg/kg/day, every 12 hours) was administrated by oral to xenograft-bearing mice. Cyclopamine was dissolved in PBS containing 10% 2-hydroxylpropyl-h-cyclodextrin (Sigma-Aldrich) at a concentration of 2.5 mg/mL. The medication was given daily for 7 days. Tumor volume was calculated as (length x width^2^) x 0.5. Xenograft tissues were then fixed with zinc-fixative solution (IHC ZINC fixative, BD Pharmingen) for 24 hours at room temperature and embedded in paraffin for immunohistochemistry.

***Bone Marrow Transplantation (BMT) Model***

Female CD1 nude mice aged 6 weeks were used as recipients and BMT was performed as reported previously [4]. BM mononuclear cells (BMMNC) from Tie2/LacZ and GFP transgenic mice were isolated by density centrifugation over Histopaque-1083 (Sigma). The recipient mice were lethally irradiated with 9.5 Gy subsequent to intraperitoneal injection of anti-asialo-GM1 antibody and intravenous injection of 2 x10^6^ BMMNCs. All tumor and matrigel implantation experiments in the chimeric mice were carried out 4 weeks after hematopoietic reconstitution. For xenograft implantation, anti-asialo-GM1 antibody was repeatedly injected every 3 day up to 7 days after tumor cell inoculation.

For the matrigel plug assay, matrigel mixed with concentrated conditioned medium (CM) from KP-1N pancreatic cancer cells or PBS was implanted subcutaneously into the back of Tie2/LacZ-BMT mice. CM was prepared by culturing KP-1N cells in serum-free medium (Ultraculture, Lonza) for 48 hours, and a centrifugal filter device (Amicon ultra-15, 5 kD, Millipore) was utilized for concentration. In the chimeric mice, endothelial/monocytic cells derived from BM express β-galactosidase through the Tie2 promoter. Growth factor-reduced matrigel (Becton Dickinson) was utilized to minimize the effect of growth factors carried in the matrigel. The animals were sacrificed 7 days after implantation, and the matrigel was removed and fixed with paraformaldehyde for immunohistochemistry.

***Histological Assessment***

For immunofluorescence staining in xenograft tissue, 4-μm sections were incubated with a CD31-specific antibody, MEC13.3 (1:50; BD Pharmingen), overnight at 4°C. Blood vessels were counted in 5-10 random viable fields (20x objective), and the vessel area/density was quantified using ImageJ software (ver 1.38). For other immunohistochemical studies the following antibodies were utilized; anti-GFP (Acris; 1:1000), anti-VE-cadherin (BD Pharmingen; 1:10), anti-smooth muscle actin (DAKO; 1:50), andti-NG2 (Chemicon; 1:200,), anti-Ptch1 (Abcam; 1:200 and SantaCruz; 1:100) and anti-Gli2 antibody (Abcam, ab26056; 1:200 and SantaCruz, G-20; 1:100). Ki-67 staining with MIB-1 antibody (DAKO; 1:100) and TUNEL staining with the ApoAlert DNA fragmentation detection kit (Clontech) were also performed. Nuclei were counterstained with 100 ng/mL DAPI (Sigma), and images were examined with a fluorescent microscope (BX-51, Olympus).

To evaluate the recruitment of BM-derived cells into subcutaneously injected matrigel, fluorescent immunostaining for β-gal was performed. Sections were incubated with rabbit polyclonal anti β-gal antibody (Chemicon; 1:1000) at 4 ℃ overnight.

***Quantitative real-time PCR assay***

Total RNA was extracted using the RNeasy Protect Mini kit (QIAGEN) according to the manufacturer’s instructions. TaqMan Gene Expression Assay primer and probe sets (Applied Biosystems) were utilized for real-time, quantitative PCR analysis. Primer sequences are summarized in **Table S1**. Transcript levels were normalized to the 18S rRNA. Results are expressed as normalized expression values (=2^-Δ^*^C^*^T^) or normalized expression relative to control cells or tissues without treated by cyclopamine or anti-Shh (=2^-ΔΔ^*^C^*^T^), unless otherwise stated. We confirmed species specificities by demonstrating flat Ct curve utilizing cDNA from human cancer cells with primers/probes for mice gene (or mice cDNA with primers/probes for human genes).

***Bone marrow mononuclear cell isolation and preparation of BM-derived pro-angiogenic cells***

Murine BMMNCs were isolated by density gradient centrifugation using Histopaque-1083 (Sigma) and cultured in EBM2 with supplements (EGM2-MV BulletKit, Lonza) and 10% FBS but without hydrocortisone on rat vitronectin (Sigma)-coated dishes [5]. Attached cells were suspended at 4 days and reseeded to new culture dish for *in vitro* studies. More than 95 % of attached cells were positive for acLDL (Biomedical Technologies) uptake and BS1 lectin (Vector laboratories) binding, confirming endothelial linage (**Figure S5**). Sixty percent of the cells express CD11b. In addition, expression of typical endothelial markers such as VE-cadherin, Flk-1 and weak expression of c-Kit was identified in the attached MNCs by flow cytometry (antibody was purchased from Beckman Coulter and BD).

***Co-culture, migration and tube formation assay***

One million mouse BM-derived proangiogenic cells (BMPCs; day-4 culture) were reseeded on 6-well plates and pretreated for 1 hour with cyclopamine in EGM2-MV medium (10 % FBS). A transwell chamber (pore size; 0.4 μm, Corning) with confluent KP-1N cells was then placed in the well and further incubated at 37°C and 5 % CO_2_ for 12h. Total RNA was extracted from the BMPCs to evaluate the effect of secreted factors from pancreatic cancer cell on gene expression in BMPCs.

Migration of the BMPCs in response to secreted factors from KP-1N was assessed by utilizing a transwell system (Corning), as described previously [4]. Briefly, 2 x10^5^ KP-1N cells were seeded on 24-well cell culture plate with regular culture medium and the medium was switched to EBM2 (0.5 % FBS) before assay. Ten thousand murine BMPCs (day 4) were reseeded on the gelatin-coated (0.1 %) transwell with polycarbonate filter (pore size; 5 μm, upper wells) and grown in EGM2 (10 % FBS) overnight and pretreated with/without cyclopamine for 1 hour. The medium was switched to EBM2 (0.5 % FBS) with/without cyclopamine and the transwell was placed on a 24-well cell culture plate with KP-1N cells (lower wells). For a positive control, the lower well was filled with medium containing 50 ng/mL human recombinant VEGF. Plates were incubated at 37 °C and 5 % CO_2_ for 18 hours. Migration activity of the BMPCs was evaluated as the mean number of migrated cells to bottom side of transwell stained with DAPI.

Experiments described above were repeated using the BMPCs knocked-down of the Smo gene using lentivirus (LV)-mediated transduction of Smo-specific shRNAs (Santa Cruz; sc-40162-V). Cells were infected either by shControl-LV or shSmo-LV in the presence of 4 μg/mL polybrene at MOI of 1.5. Transduction efficiency as monitored by fluorescent microscopy at 48 h post-infection of GFP-LV was 70-80 %.

Tube formation assay was performed by co-culturing BMPCs with mice endothelial cells MS-1 on matrigel as descrived previously [6].

***Statistical analysis***

All results are expressed as mean ± standard deviation unless otherwise noted. Statistical significance of differences was determined using a two-tailed Student’s t-test.

**References**

1. Nakano Y, Tanno S, Koizumi K, Nishikawa T, Nakamura K, et al. (2007) Gemcitabine chemoresistance and molecular markers associated with gemcitabine transport and metabolism in human pancreatic cancer cells. Br J Cancer 96: 457-463.

2. Thayer SP, di Magliano MP, Heiser PW, Nielsen CM, Roberts DJ, et al. (2003) Hedgehog is an early and late mediator of pancreatic cancer tumorigenesis. Nature 425: 851-856.

3. Feldmann G, Dhara S, Fendrich V, Bedja D, Beaty R, et al. (2007) Blockade of hedgehog signaling inhibits pancreatic cancer invasion and metastases: a new paradigm for combination therapy in solid cancers. Cancer Res 67: 2187-2196.

4. Ii M, Takenaka H, Asai J, Ibusuki K, Mizukami Y, et al. (2006) Endothelial progenitor thrombospondin-1 mediates diabetes-induced delay in reendothelialization following arterial injury. Circ Res 98: 697-704.

5. Ii M, Nishimura H, Iwakura A, Wecker A, Eaton E, et al. (2005) Endothelial progenitor cells are rapidly recruited to myocardium and mediate protective effect of ischemic preconditioning via "imported" nitric oxide synthase activity. Circulation 111: 1114-1120.

6. Yamazaki M, Nakamura K, Mizukami Y, Ii M, Sasajima J, et al. (2008) Sonic hedgehog derived from human pancreatic cancer cells augments angiogenic function of endothelial progenitor cells. Cancer Sci 99: 1131-1138.
