# Supplementary figures and images for: Hedgehog Promotes Neovascularization in Pancreatic Cancers by Regulating Ang-1 and IGF-1 Expression in Bone-Marrow Derived Pro-Angiogenic Cells
Source: PLoS One. 2010 Jan 21;5(1):e8824. doi: 10.1371/journal.pone.0008824 (PMC2809097; doi:10.1371/journal.pone.0008824)

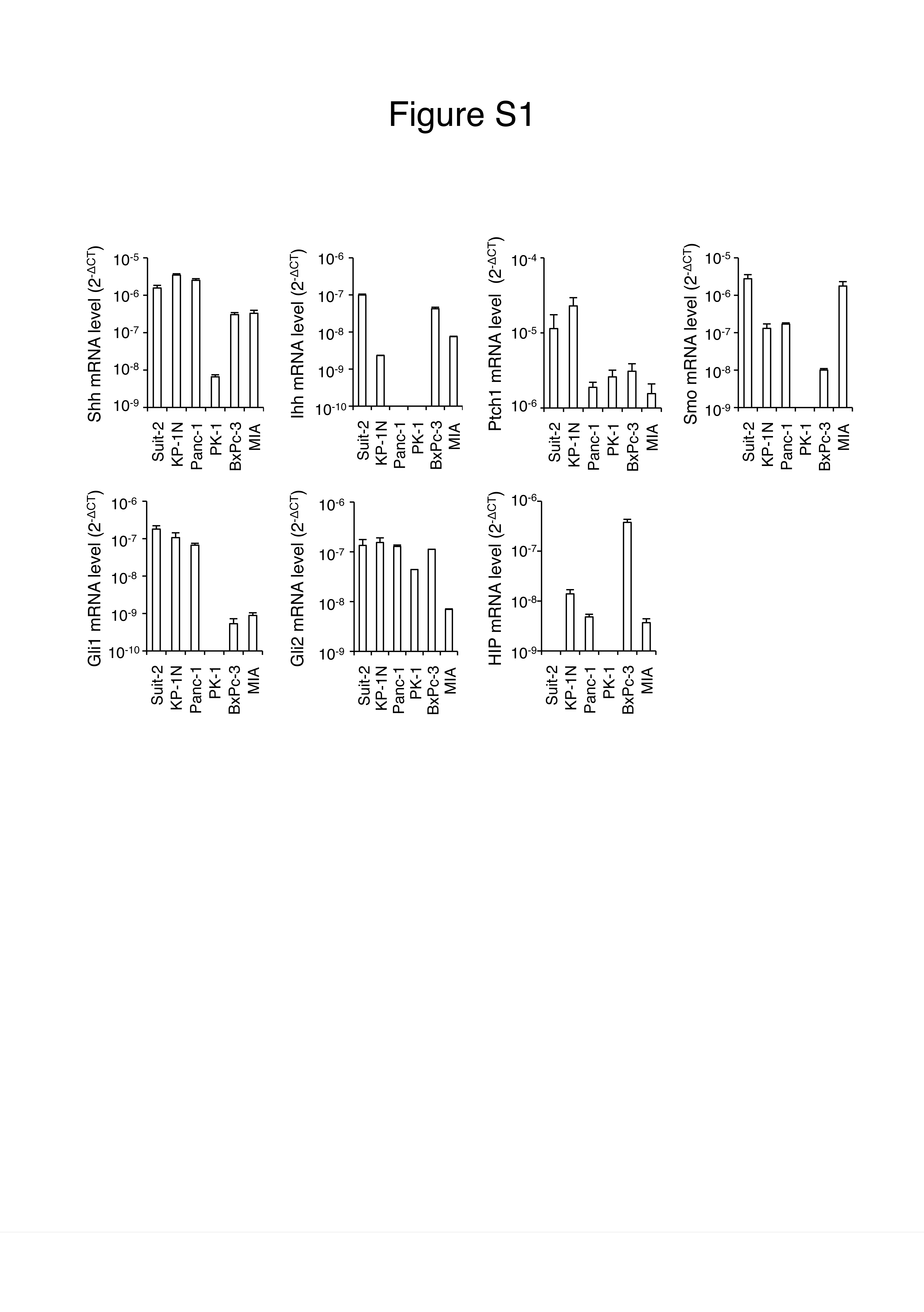

Supplement: Figure S1 — Expression of Hh signaling components. Quantitative RT-PCR profiling of Hh pathway genes was assessed by Taqman assay. (0.73 MB TIF) [file pone.0008824.s002.tif]

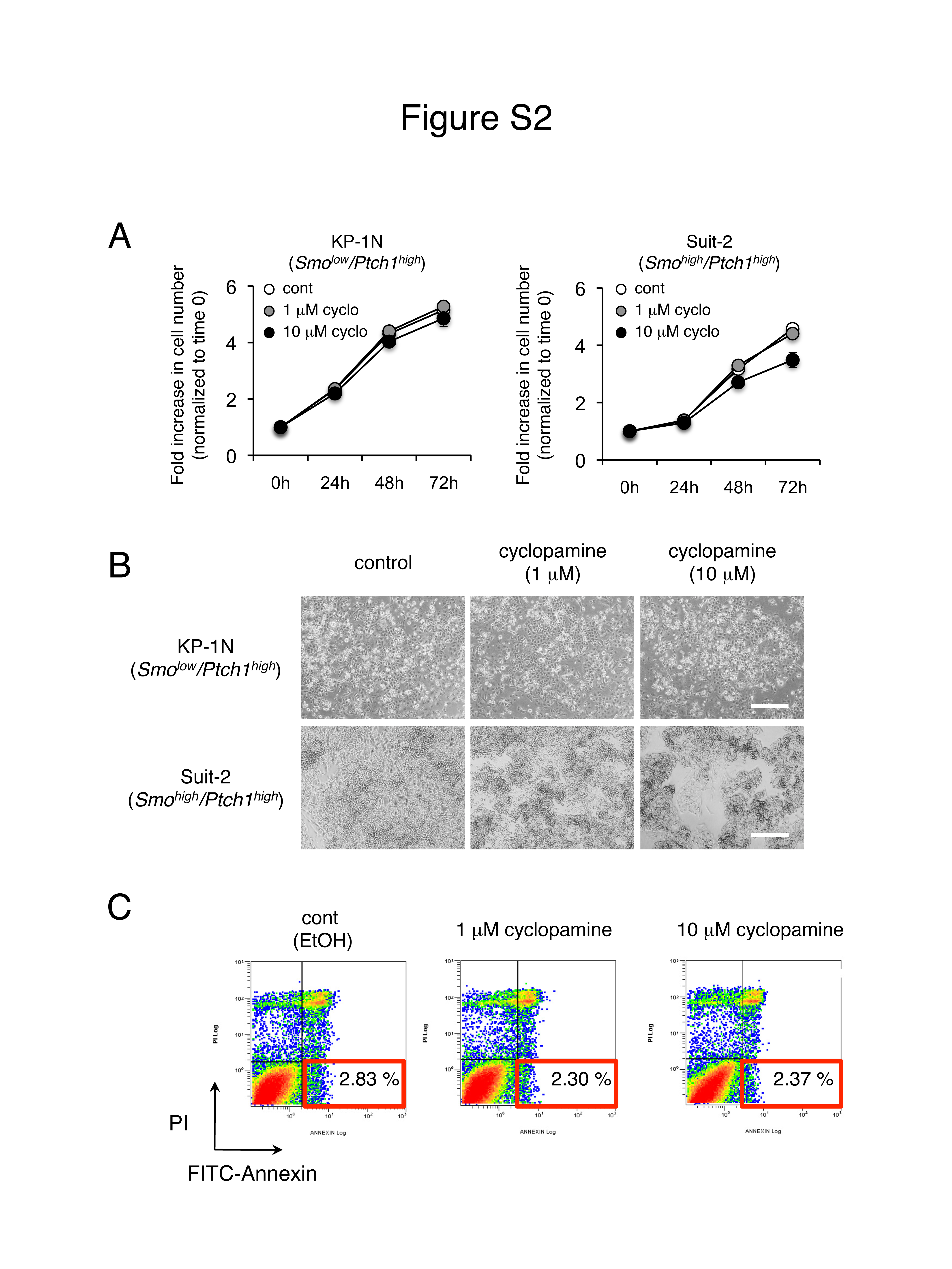

Supplement: Figure S2 — Effects of cyclopamine on human PDAC cell proliferation/death in vitro. (A) Cell proliferation assay was performed in the presence of cyclopamine. (B) KP-1N and Suit-2 cells were cultured with cyclopamine for 7 days. The culture medium containing fresh cyclopamine was changed every 2 days. (C) Annexin V flow cytometry analysis. KP-1N cells were treated with 1–10 µM cyclopamine for 24 hours. The early apoptotic fraction was quantified as FITC-Annexin V+/PI- fraction (indicated by red frame). (5.75 MB TIF) [file pone.0008824.s003.tif]

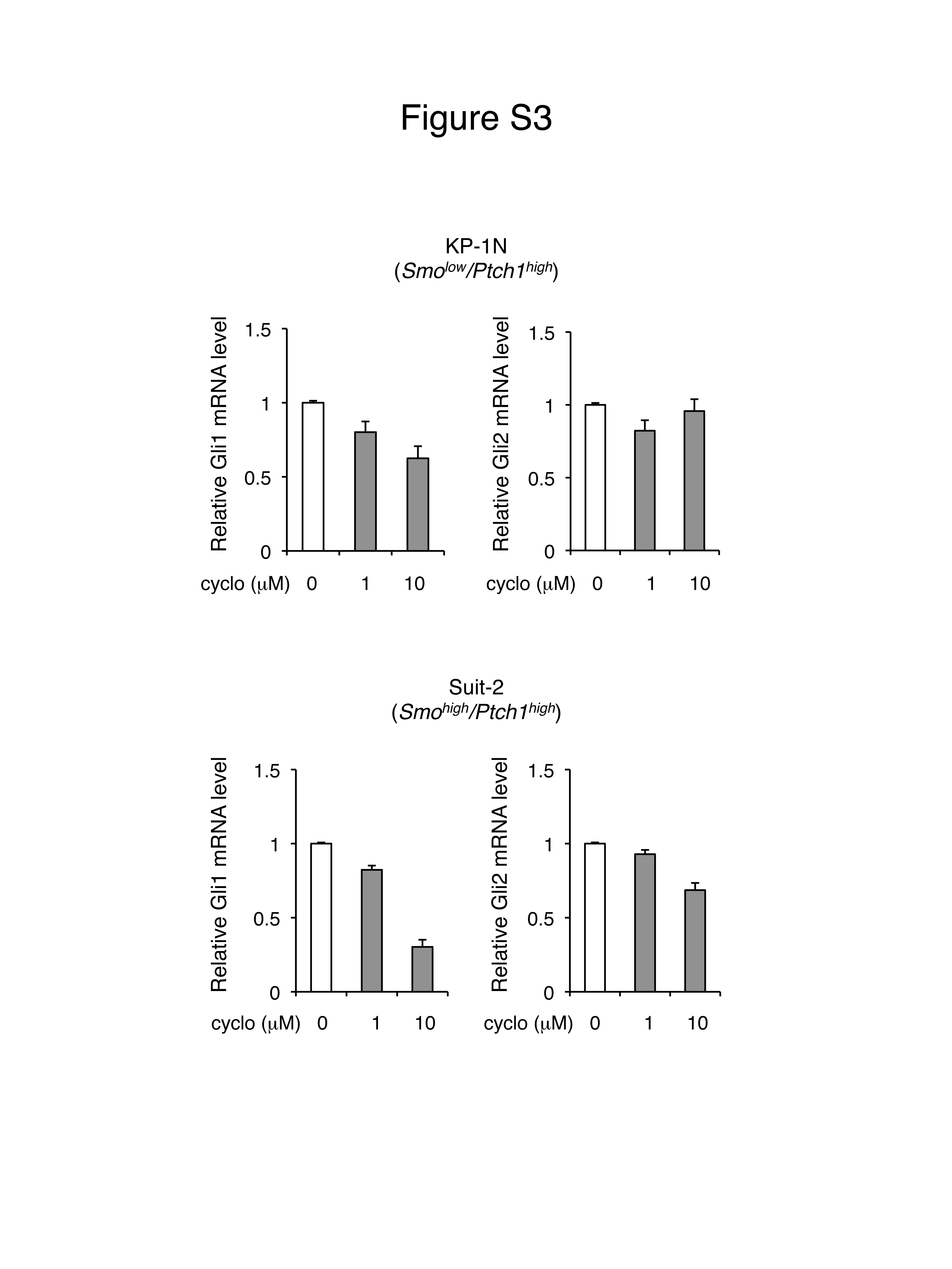

Supplement: Figure S3 — Reduction of Gli1/Gli2 mRNA levels by cyclopamine in pancreatic cancer cells in vitro. KP-1N and Suit-2 cells were treated with 1–10 µM of cyclopamine (cyclo) for 8 hours and Gli1 and Gli2 mRNA expression was quantified. (0.51 MB TIF) [file pone.0008824.s004.tif]

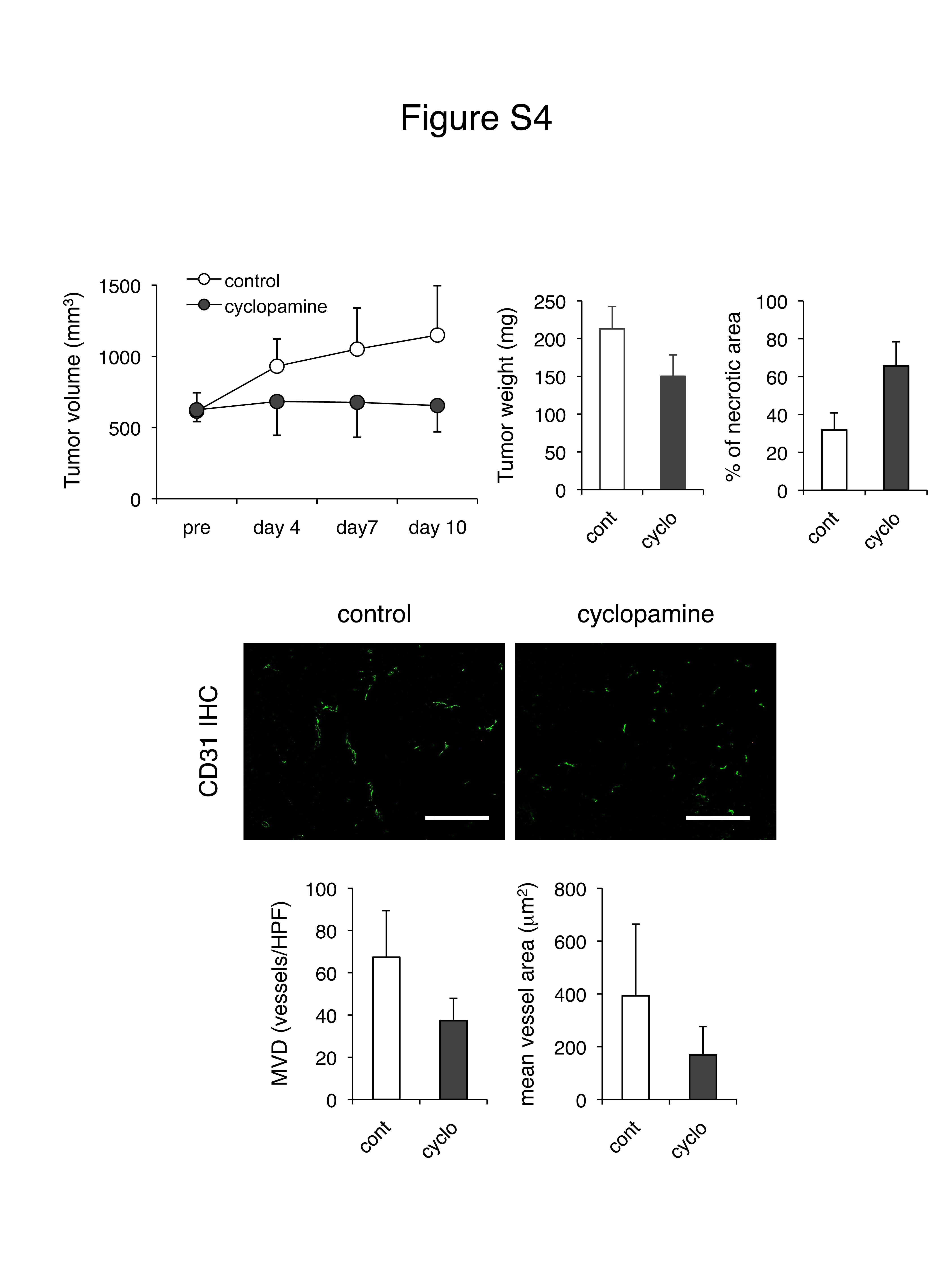

Supplement: Figure S4 — Cyclopamine inhibits growth and tumor angiogenesis of Suit-2 xenogtafts. CD-1 nude mice bearing Suit-2 xenografts were treated with or without cyclopamine (50 mg/kg/day, dissolved in PBS containing 10% 2-hydroxylpropyl-h-cyclodextrin at a concentration of 2.5 mg/mL) administrated by oral daily for 7 days (6 xenografts for each group). The results are shown as mean ± SEM tumor weight (mg). Sections were stained with anti-CD31. Scale bars; 500 µm. The data are shown as mean ± SEM MVD and mean vessel area. (0.88 MB TIF) [file pone.0008824.s005.tif]

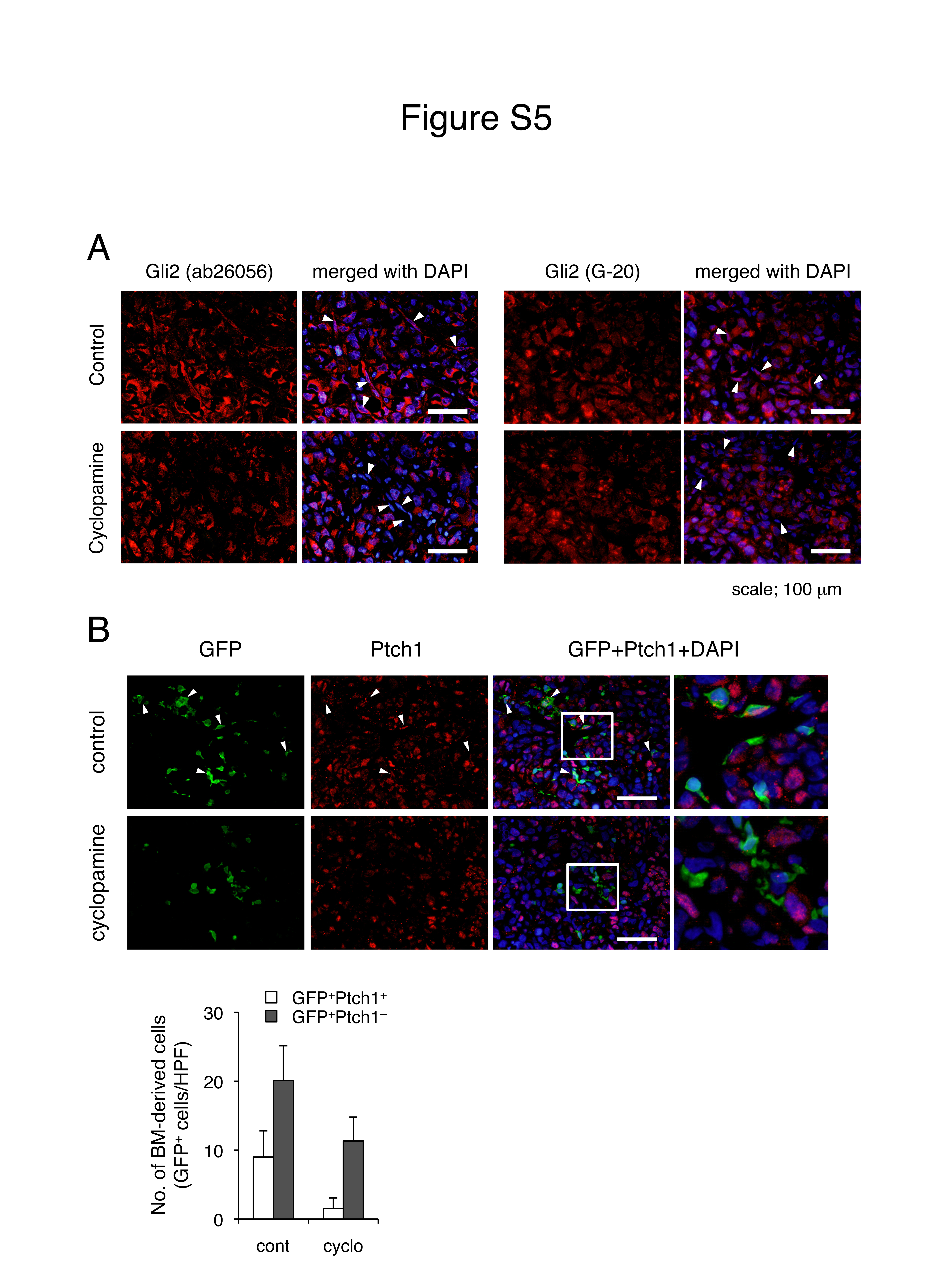

Supplement: Figure S5 — Downregulation of full length Gli2 and Ptch1 expression in stroma by Hedgehog blockade. (A) KP-1N xenografts treated with or without cyclopamine were stained with anti-Gli2 antibody (Abcam, ab26056 and SantaCruz, G-20). (B) KP-1N xenograft tissues on GFP-BMT-chimeric mice were immunostained with Ptch1 in combination with GFP. The results shown are mean number of GFP+ BM-derived cells positive and negative for Ptch1. Scale bars; 100 µm. (9.54 MB TIF) [file pone.0008824.s006.tif]

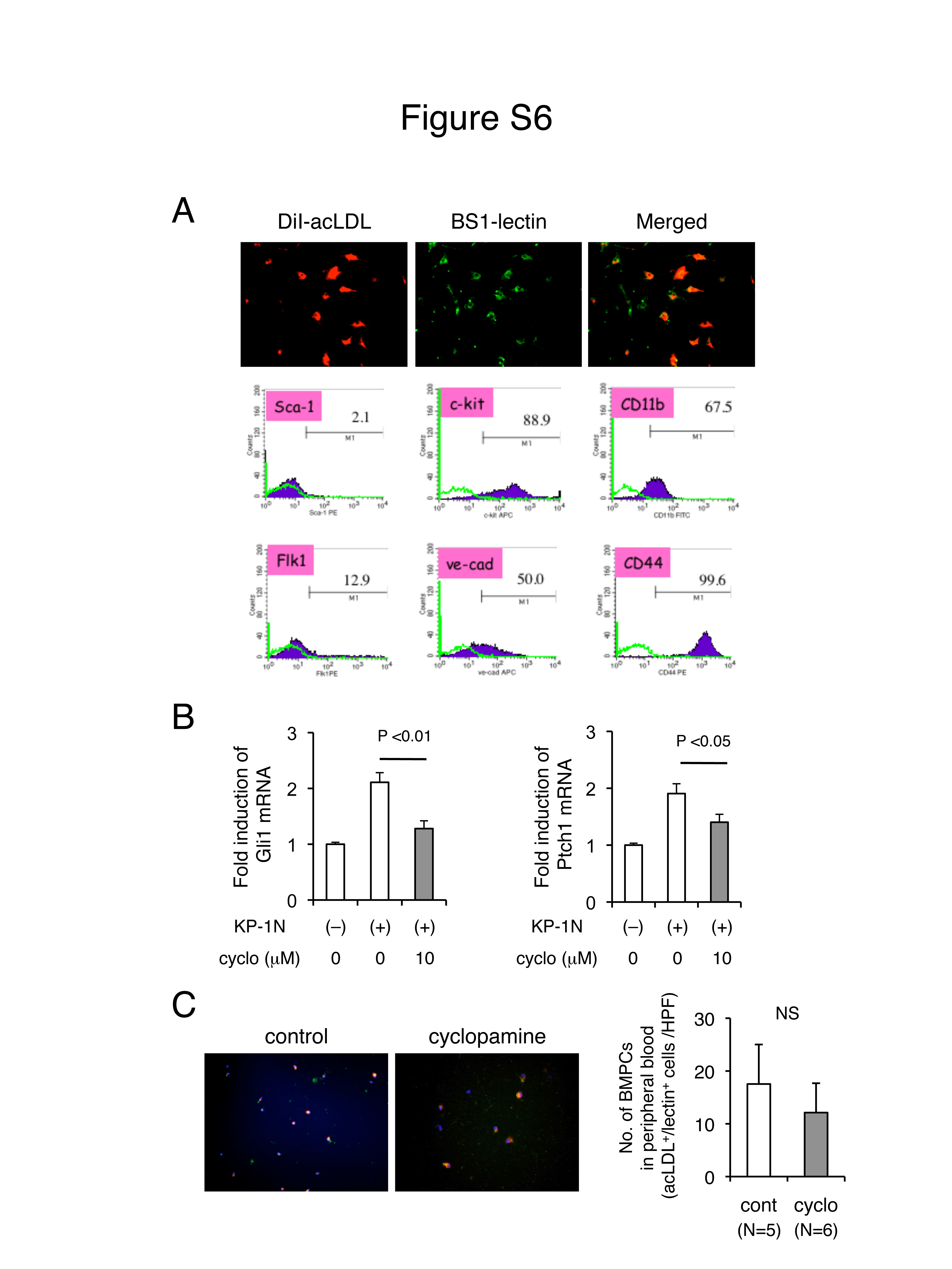

Supplement: Figure S6 — Characterization of cultured BM-derived pro-angiogenic cells. BMMNCs cultured with EGM2-MV medium (10% FBS) in vitronectin-coated dish were utilized as BM-derived pro-angiogenic cells (BMPCs) for in vitro experiments. (A) After 4–7 days culture, spindle shaped attached cells were incubated with acetylated LDL (acLDL; DiI labeled) and labeled with BS1-lectin (FITC-conjugated). Flow cytometric analysis of attached cells on day 7. Data show the percentage of positive cells for progenitor and endothelial markers. (B) BMPCs were seeded in the lower well and co-cultured with KP-1N cells in the upper well in the presence or absence of 10 µM cyclopamine. RNA was harvested from the BMPCs 12 h after for qPCR analysis (normalized to the cells without KP-1N). (C) Circulating BMPCs in KP-1N xenograft-bearing mice are quantified. MNCs from 500 µL blood were cultured with EGM2-MV medium for 4 days and labeled with DiI-acLDL/FITC-isolectin B4. The results shown as the mean ± SEM number of circulating BMPCs (double positive cells/field). (1.98 MB TIF) [file pone.0008824.s007.tif]

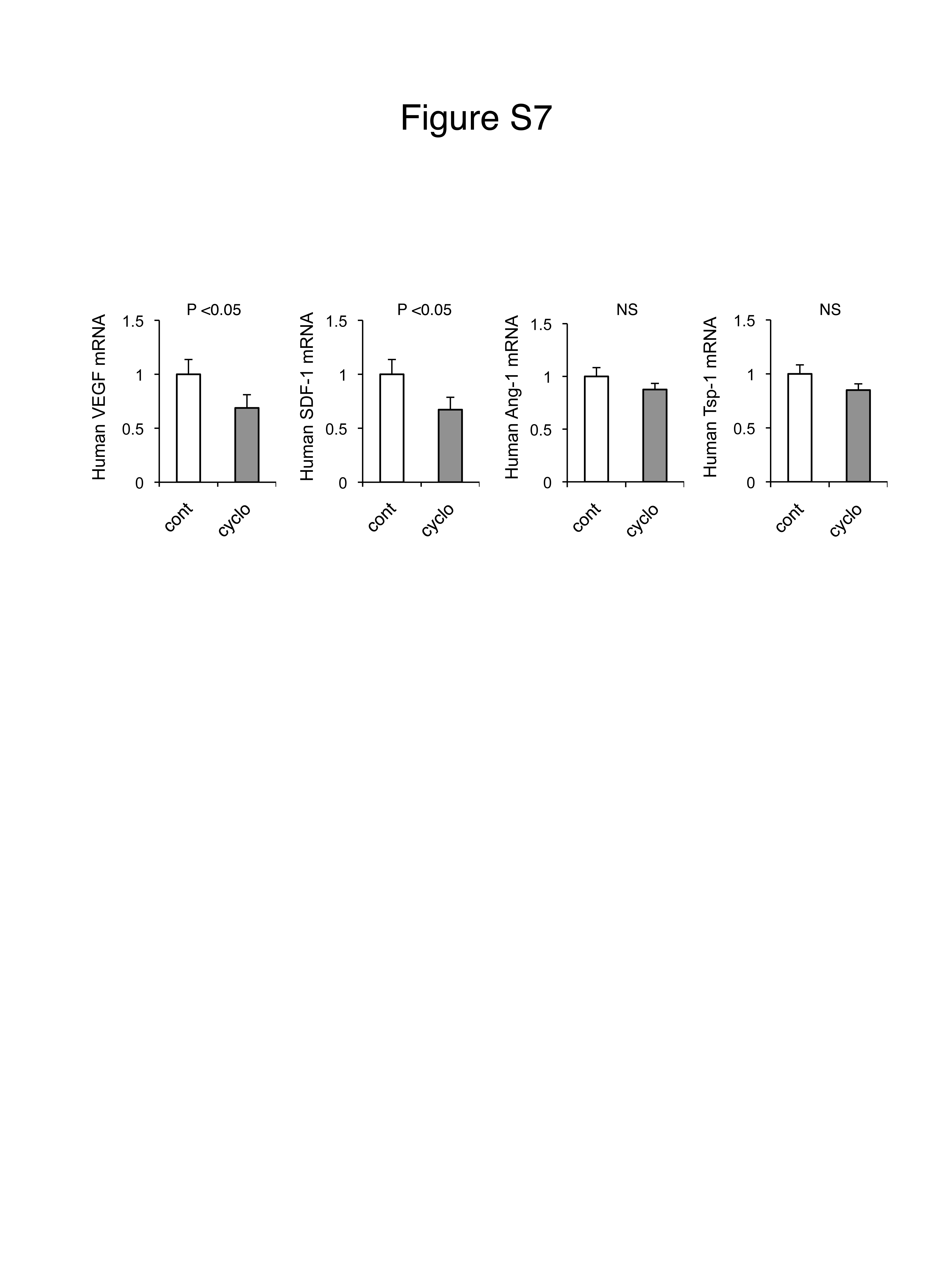

Supplement: Figure S7 — Cyclopamione downregulate human VEGF and SDF-1 in KP-1N xenografts. mRNA expressions of human pro-/anti-angiogenic factors in xenografts were quantified by qPCR. (0.41 MB TIF) [file pone.0008824.s008.tif]

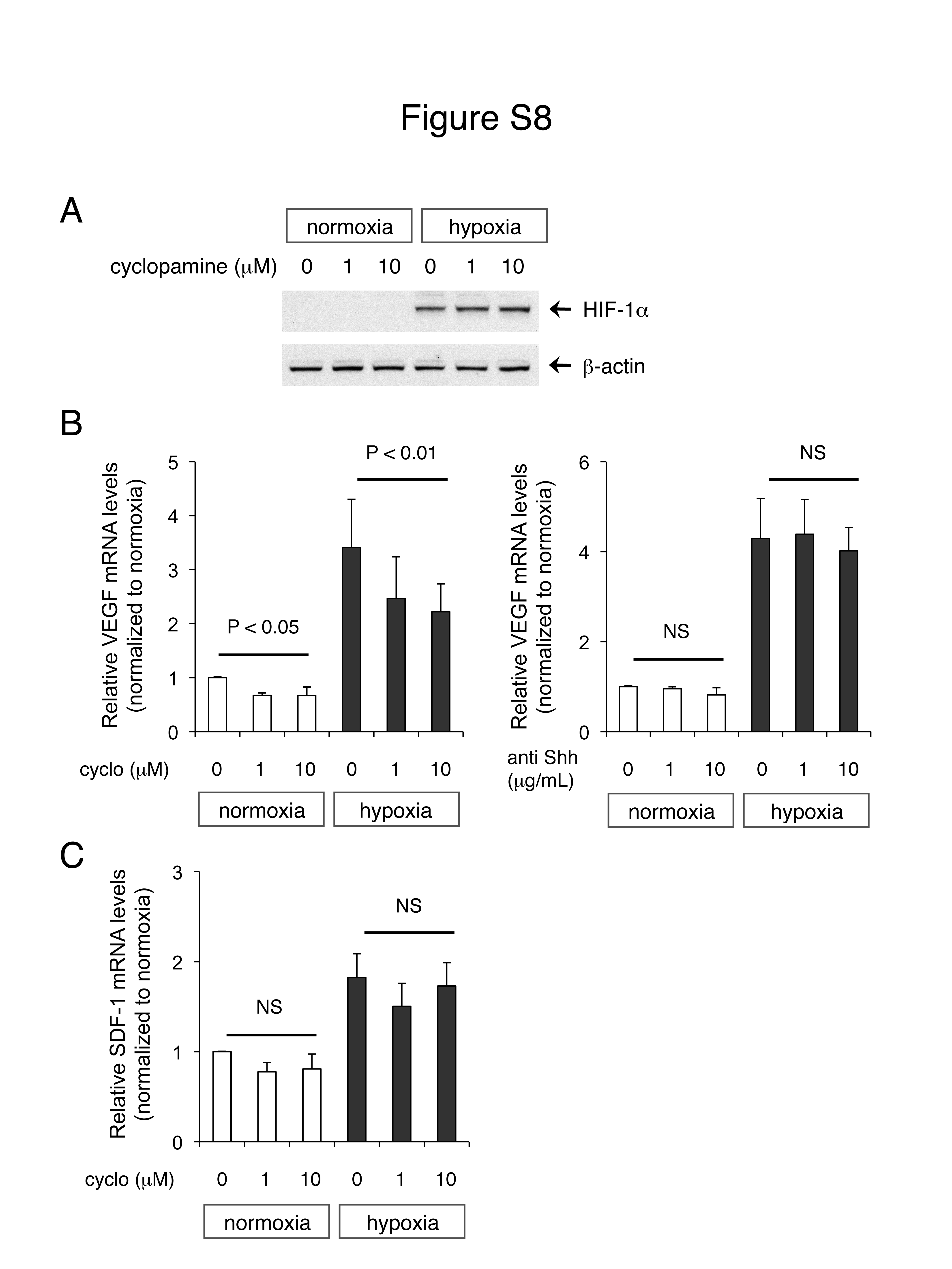

Supplement: Figure S8 — Hedgehog blockade does not reduce VEGF and SDF-1 mRNA expression in KP-1N cells in vitro. (A) KP-1N were treated with 1–10 µM cyclopamine for 1 h and then cultured either in normoxic (20% O2) or hypoxic (1% O2) conditions. Protein lysates were harvested after 8 h incubation to detect HIF-1α protein by western blotting. Immunoblot analysis for anti-HIF-1α (clone 54, 1∶250, BD) and β-actin (1∶5000, Sigma) was performed. (B, C) Total RNA was extracted after 8 h incubation with 1–10 µM cyclopamine or 1–10 µg/mL anti-Shh (MAB4641; R&D systems) under normoxic or hypoxic conditions to quantify VEGF and SDF-1 mRNA by qPCR. (1.10 MB TIF) [file pone.0008824.s009.tif]

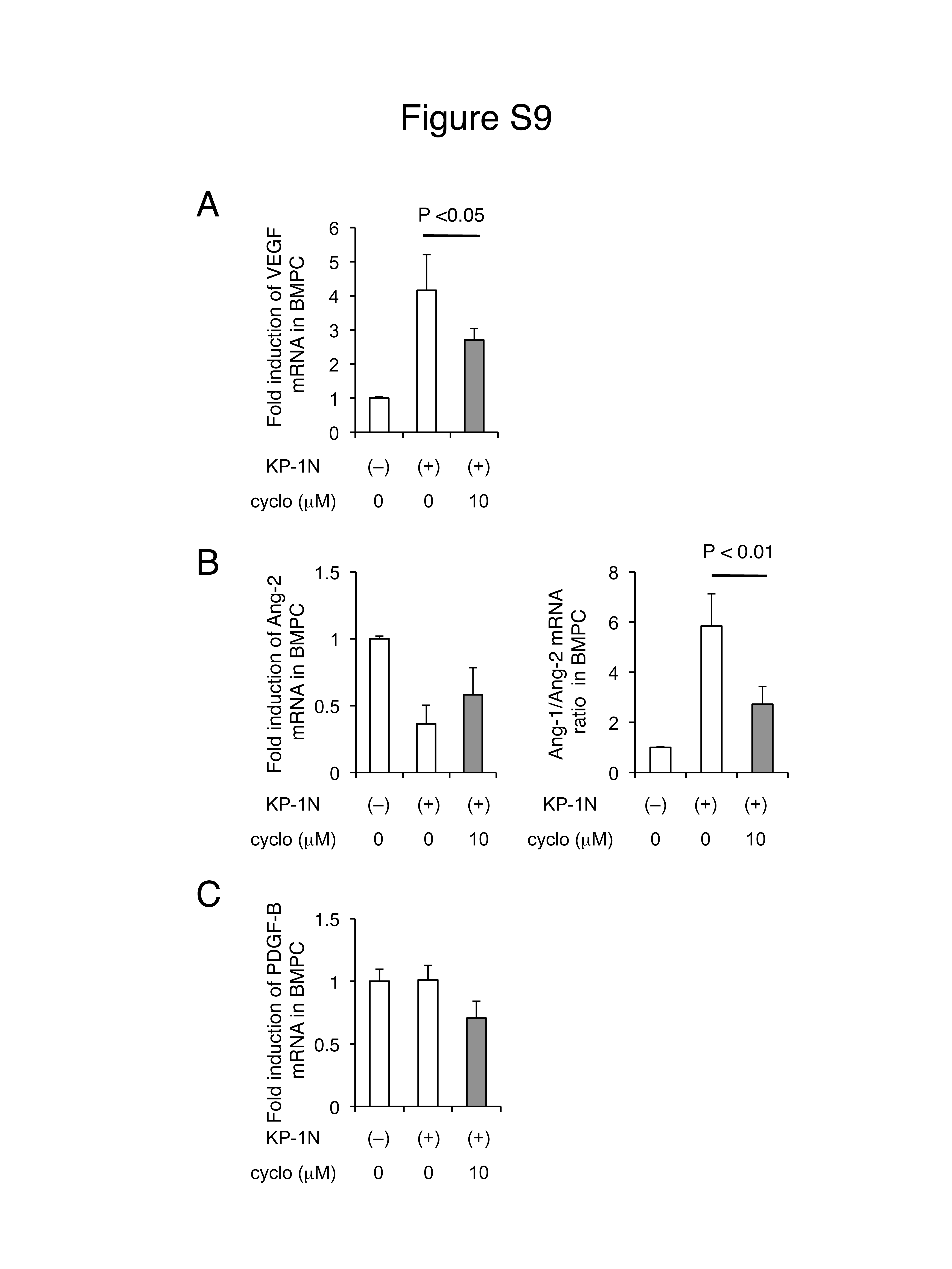

Supplement: Figure S9 — VEGF, Ang-2 and PDGF-B expression in BM-derived pro-angiogenic cells was downregulated by cyclopamine. (A and B) Mouse BM-derived pro-angiogenic cells (BMPCs) were co-cultured with KP-1N utilizing transwell (0.4 µm pore). BMPCs were seeded on lower well and KP-1N cells on upper well. mRNA levels for mouse VEGF (A) Ang-1/Ang-2 (B), and PDGF-B (C) in the BMPCs were quantified by qPCR after 12 h co-culture with or without 10 µM cyclopamine (normalized to the BMPCs without KP-1N). (0.52 MB TIF) [file pone.0008824.s010.tif]

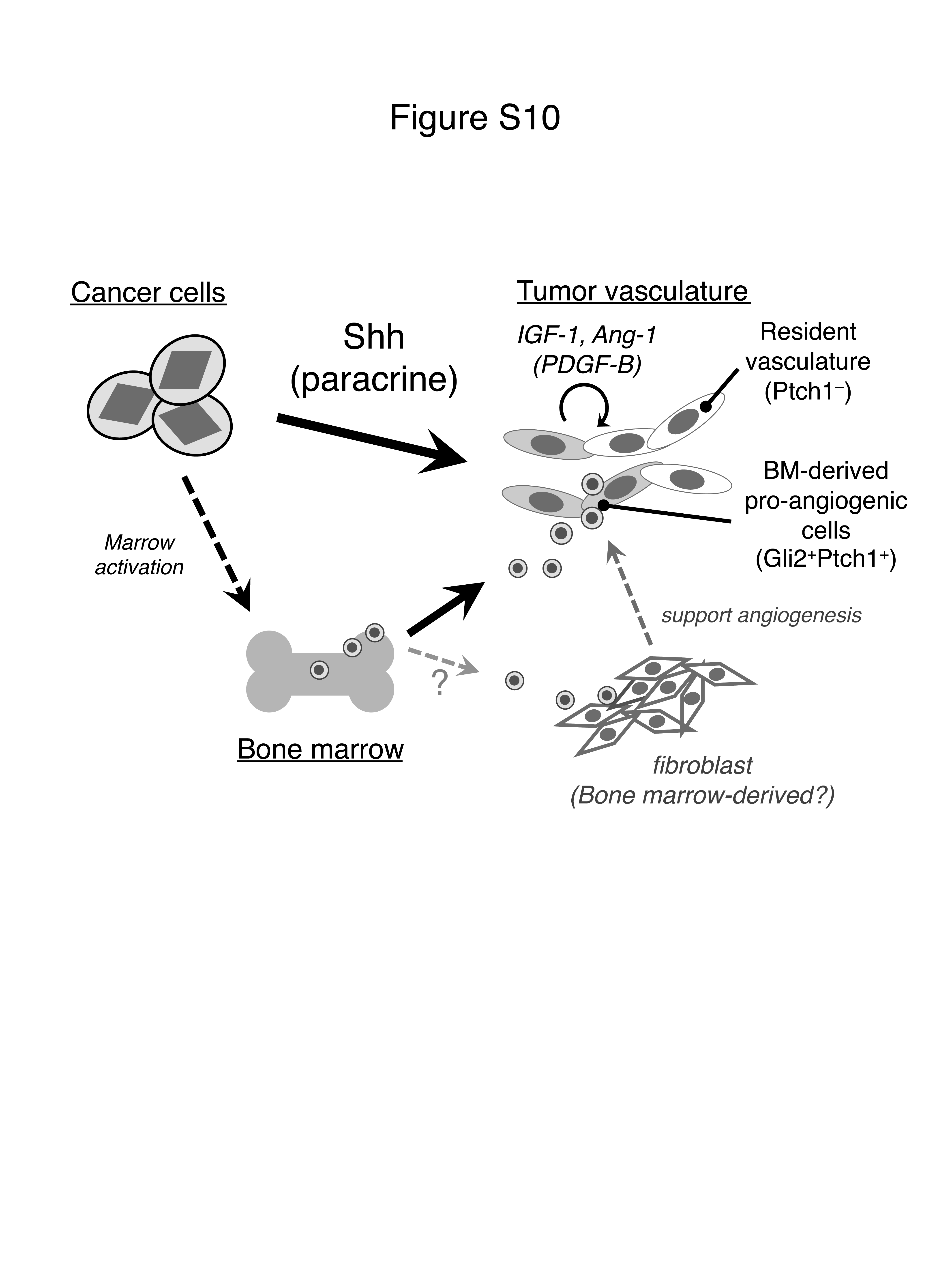

Supplement: Figure S10 — Proposed model for Hh-mediated angiogenesis in PDAC. Shh is an early mediator for pancreatic tumorigenesis, but it may not be sufficient for proliferation of cancer cells at late stages. The paracrine effect of Hh emerges during tumorigenesis by acting through BM-derived cells including pro-agiogenic (precursor) cells and contributing to the development and maintenance of the tumor vasculature. Hh could also contribute to the activation of stromal fibroblasts (potentially derived from the BM) that potentially promote tumorigenesis through supporting angiogenesis or other mechanisms. (0.74 MB TIF) [file pone.0008824.s011.tif]
