# Supplementary material for: Hedgehog Promotes Neovascularization in Pancreatic Cancers by Regulating Ang-1 and IGF-1 Expression in Bone-Marrow Derived Pro-Angiogenic Cells
Source: PLoS One. 2010 Jan 21;5(1):e8824. doi: 10.1371/journal.pone.0008824 (PMC2809097; doi:10.1371/journal.pone.0008824)
Supplement: Table S1 — (0.05 MB DOC) [file pone.0008824.s012.doc]

**Supplementary Table 1.** Primer sequences used for quantitative RT-PCR assays.

TaqMan® Gene Expression Assays (Applied Biosystems) were used as follows:

|  | Primer sequences used for quantitative RT-PCR assays | | | |
| --- | --- | --- | --- | --- |
|  | TaqMan® Gene Expression Assays (Applied Biosystems) were used as follows: | | | |
|  |  |  |  |  |
|  | *human* |  | *mouse* |  |
|  |  |  |  |  |
|  | Shh | Hs00179843_m1 | Ptch1 | Mm00436014_m1 |
|  | Ihh | Hs01081800_m1 | Smo | Mm01162710_m1 |
|  | Smo | Hs00170665_m1 | Gli1 | Mm00494654_m1 |
|  | Ptch1 | Hs00970980_m1 | Gli2 | Mm01293117_m1 |
|  | Gli1 | Hs01110766_m1 | HIP | Mm00469580_m1 |
|  | Gli2 | Hs00257977_m1 | VEGF | Mm00437304_m1 |
|  | HIP | Hs01011008_m1 | SDF-1 | Mm00445552_m1 |
|  | VEGF | Hs00900054_m1 | Ang-1 | Mm00456498_m1 |
|  | SDF-1 | Hs00930455_m1 | Ang-2 | Mm00545822_m1 |
|  | Ang-1 | Hs00375823_m1 | IGF-1 | Mm00439560_m1 |
|  | IGF-1 | Hs01547656_m1 | PDGF-B | Mm01298578_m1 |
|  | Tsp-1 | Hs00170236_m1 | Tsp-1 | Mm00449022_m1 |
|  |  |  |  |  |
|  | 18S rRNA | Hs99999901_s1 (used as control in all assays) | | |
|  |  |  |  |  |
|  |  |  |  |  |
