# Supplementary material for: Hedgehog Promotes Neovascularization in Pancreatic Cancers by Regulating Ang-1 and IGF-1 Expression in Bone-Marrow Derived Pro-Angiogenic Cells
Source: PLoS One. 2010 Jan 21;5(1):e8824. doi: 10.1371/journal.pone.0008824 (PMC2809097; doi:10.1371/journal.pone.0008824)
Supplement: Table S2 — (0.04 MB DOC) [file pone.0008824.s013.doc]

**Supplementary Table 2.** Growth inhibition by cyclopamine in human PDAC lines.

Reduction in the number of PDAC cells by cyclopamine (10 μM) treatment was evaluated by a cell proliferation assay kit, WST-8 (Quick Cell Proliferation Assay Kit, Biovision). Cells were cultured in the presence or absence of cyclopamine for 72 hours.

| % reduction in number of cells in the presence of 10 μM cyclopamine | | |
| --- | --- | --- |
| (WST-8 assays were performed to quantify growth inhibition at day3.) | | |
|  |  |  |
| Cell line |  | % reduction |
| Suit-2 | *Smo^high^/Ptch1^high^* | 23.8 ± 0.26 |
| KP-1N | *Smo^low^/Ptch1^high^* | 5.7 ± 0.29 |
| Panc-1 | *Smo^low^/Ptch1^low^* | -0.6 ± 0.05 |
| PK-1 | *Smo^low^/Ptch1^low^* | 3.6 ± 0.38 |
| BxPC-3 | *Smo^low^/Ptch1^low^* | 13.3 ± 1.14 |
| MIA-PaCa2 | *Smo^high^/Ptch1^low^* | 23.9 ± 0.08 |
